# Supplementary material for: Cis-regulatory control of transcriptional timing and noise in response to estrogen
Source: Cell Genom. 2024 Apr 24;4(5):100542. doi: 10.1016/j.xgen.2024.100542 (PMC11099348; doi:10.1016/j.xgen.2024.100542)
Supplement: Document S2. Article plus supplemental information [file mmc2.pdf]

Cell Genomics

Article

Cis-regulatory control of transcriptional timing and noise in response to estrogen

Graphical abstract

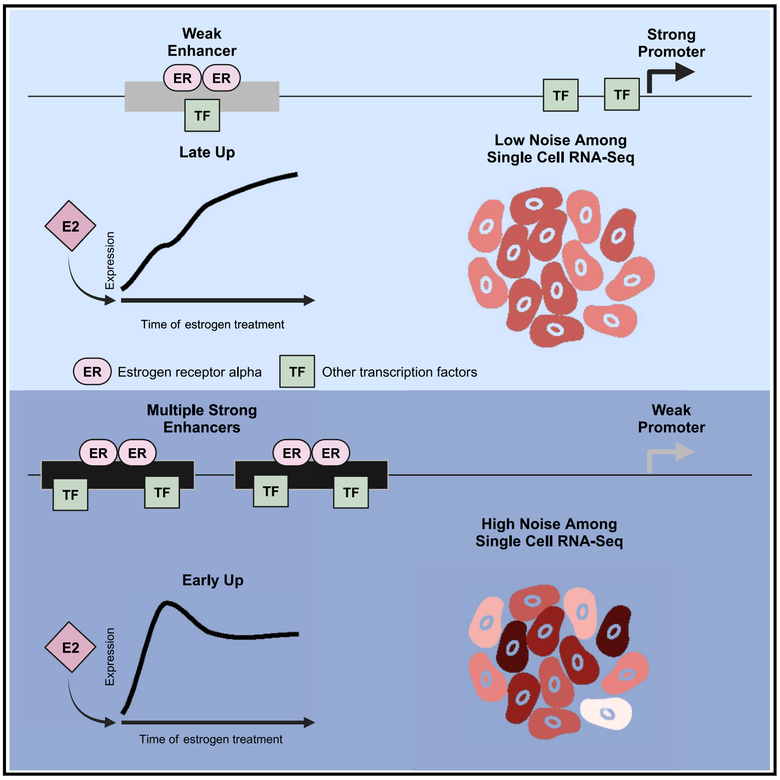

Highlights

- Analysis of estrogen transcriptional responses over time in single human cells
- Multiple estrogen-receptor-bound enhancers associate with fast responses
- Temporal responses can be altered through modulation of enhancer activity
- Enhancer-driven genes exhibit higher noise than promoter-driven genes

Authors

Matthew Ginley-Hidinger, Hosiana Abewe, Kyle Osborne, ..., John Lis, Xiaoyang Zhang, Jason Gertz

Correspondence

jay.gertz@hci.utah.edu

In brief

Through temporally resolved single-cell analysis of transcriptional responses to estrogen, Ginley-Hidinger et al. discovered that the presence of multiple estrogen-receptor alpha-bound enhancers is associated with fast responses to estrogen and that epigenetic modulation of enhancer activity can change the temporal response. They also found that enhancer-driven genes exhibit high transcriptional noise compared with promoter-driven genes.

Ginley-Hidinger et al., 2024, Cell Genomics *4*, 100542
May 8, 2024 © 2024 The Author(s). Published by Elsevier Inc.
https://doi.org/10.1016/j.xgen.2024.100542

CellPress

Cell Genomics

CellPress
OPEN ACCESS

Article

Cis-regulatory control of transcriptional timing and noise in response to estrogen

Matthew Ginley-Hidinger,[1,2] Hosiana Abewe,[1,3] Kyle Osborne,[1,3] Alexandra Richey,[1,2] Noel Kitchen,[1,3] Katelyn L. Mortenson,[1,3] Erin M. Wissink,[4] John Lis,[4] Xiaoyang Zhang,[1,3] and Jason Gertz[1,2,3,5,*]
[1]Huntsman Cancer Institute, University of Utah, Salt Lake City, UT 84112, USA
[2]Department of Biomedical Engineering, University of Utah, Salt Lake City, UT 84112, USA
[3]Department of Oncological Sciences, University of Utah, Salt Lake City, UT 84112, USA
[4]Department of Molecular Biology and Genetics, Cornell University, Ithaca, NY 14853, USA
[5]Lead contact
*Correspondence: jay.gertz@hci.utah.edu
https://doi.org/10.1016/j.xgen.2024.100542

SUMMARY

Cis-regulatory elements control transcription levels, temporal dynamics, and cell-cell variation or transcriptional noise. However, the combination of regulatory features that control these different attributes is not fully understood. Here, we used single-cell RNA-seq during an estrogen treatment time course and machine learning to identify predictors of expression timing and noise. We found that genes with multiple active enhancers exhibit faster temporal responses. We verified this finding by showing that manipulation of enhancer activity changes the temporal response of estrogen target genes. Analysis of transcriptional noise uncovered a relationship between promoter and enhancer activity, with active promoters associated with low noise and active enhancers linked to high noise. Finally, we observed that co-expression across single cells is an emergent property associated with chromatin looping, timing, and noise. Overall, our results indicate a fundamental tradeoff between a gene's ability to quickly respond to incoming signals and maintain low variation across cells.

INTRODUCTION

Cis-regulatory elements (CREs) control the precise spatiotemporal expression of genes across the genome. In addition to a gene's promoter, many enhancers collaborate to control a single gene's expression in mammalian cells.[1–3] External chemical signals often induce changes in cell phenotypes by altering transcription, requiring coordinated gene expression programs. Signal transduction can lead to transcription factor (TF) binding changes and epigenetic modifications at CREs.[4] For cells to appropriately respond to stimuli, CREs must guide the amount of transcript produced,[4] the timing of transcriptional changes,[5,6] and the amount of transcriptional variation or noise.[6–8] While there has been extensive research on the role that CREs play in transcription levels, less is understood about the properties of CREs that control gene expression timing and noise.

Temporal regulation of gene expression is an essential attribute of transcriptional control for cellular processes such as cell fate transitions[9–11] and responses to signals.[12–14] Specific genes, often termed immediate-early genes, are rapidly activated in response to a signal, while other genes change expression more gradually.[14–16] Genes that show coordinated trajectories are often functionally related, driving diverse phenotypes at different timescales.[13,17–19] Previous studies have identified several mechanisms that regulate transcriptional timing. One influential factor is the state of a gene's promoter. For example, pre-loading of RNA polymerase II (RNAPII) at the promoter is indicative of earlier gene expression responses.[20] Additional promoter features associated with early-responding genes include TATA motifs at the promoter, a greater number of TF binding motifs, and increased chromatin accessibility.[20,21] Enhancers are also crucial for gene expression timing. Inhibition or deletion of specific enhancers can prolong the time needed for a gene to reach maximal expression without altering final expression levels.[22,23] Stretches of potent enhancers, called super-enhancers, regulate some immediate-early genes.[24] In contrast, enhancers marked by repressive chromatin marks, termed latent enhancers, exhibit slower activation and are associated with late-responding genes.[25] Overall, relatively little is known about which genomic features in a gene's *cis*-regulatory repertoire are important for influencing stimulus-dependent temporal gene expression responses.

In addition to regulating gene expression timing and levels, CREs control the amount of transcriptional noise. Transcriptional noise is a combination of intrinsic stochasticity and extrinsic variability that cause transcript variation across a population of isogenic cells.[2,26,27] Cells must regulate transcriptional variation, as both high and low variation have functional consequences. High variation can have benefits, as cells may be more adaptable to changing environments[28,29] and more likely to undergo cell fate transitions.[30,31] Noise may additionally confer the ability of a cell population to produce a diverse output to a single incoming signal.[32] However, noise can be associated with negative consequences, such as worse cancer outcomes,[33] cancer therapy

This is an open access article under the CC BY license (http://creativecommons.org/licenses/by/4.0/).

resistance,[34,35] and the ability of cancer cells to metastasize.[36,37] Both promoters and enhancers can regulate intrinsic noise kinetics and sensitivity to extrinsic noise sources.[38] For example, nucleosome positioning and histone modifications at the promoter are important noise regulators,[27,39–42] with active histone marks at promoters often associated with low noise.[43] Additionally, a greater number of TFs binding at a promoter may be a basis for greater amounts of noise.[44] The role of enhancers in controlling mammalian expression noise is less clear. Thermodynamic modeling approaches suggest that multiple enhancers should buffer noise,[45] while experimental evidence shows that super-enhancers are generally associated with noisier expression.[27,46] A remaining challenge is understanding the effects of multiple enhancers in combination with a promoter on expression noise.

To investigate the regulatory control of timing and noise in depth, we focused on the transcriptional response to estrogens. Estrogen receptor α (ER) is a nuclear hormone receptor activated by estrogens, including endogenously produced 17β-estradiol (E2). In the presence of E2, ER becomes an active TF and regulates the expression of hundreds of genes.[47] ER is a clinically relevant TF, a potent oncogenic driver for endometrial and breast cancer,[48,49] and a well-studied model TF. Upon activation, ER both upregulates and downregulates genes at different timescales.[50,51] Following an estrogen induction, ER activates successive sets of functionally unique genes, as seen in genes related to vascularization, signaling, proliferation, and cell cycle.[19,52] ER has also been shown to regulate transcriptional noise. Live cell imaging of ER target genes *GREB1*[53] and *TFF1*[54] show that ER impacts transcriptional noise by modulating transcription kinetics. The temporal, heterogeneous complexity of the ER transcriptional program makes it an ideal model system for studying how CREs regulate transcriptional timing and noise in response to an external stimulus.

To better understand the genomic underpinnings of transcriptional levels, timing, and noise, we analyzed the transcriptional response to E2 using a time course of single-cell RNA-seq (scRNA-seq) in two cell types (human breast and endometrial cancer cells). Feature ranking approaches, using genomic data, revealed important determinants that control these transcriptional attributes. A strong enhancer repertoire was associated with earlier changes in gene expression, which was confirmed using functional perturbation by dCas9-based synthetic TFs. Promoter features also regulate timing, such as transcriptional repressor SIN3A being found at the promoters of Late genes. We uncovered a balance between enhancers and promoters in regulating expression noise, where strong enhancers drive higher noise and strong promoters are associated with low expression variance. The role of enhancers in timing and noise reveals a tradeoff between expression noise and the ability to respond quickly to incoming signals.

RESULTS

Machine learning approach accurately predicts genomic determinants of expression levels

To uncover features of gene regulation that control expression levels, timing, and noise, pooled scRNA-seq was conducted following 0, 2, 4, and 8 h of E2 treatments in two cell lines: Ishikawa (human endometrial adenocarcinoma) and T-47D (human breast carcinoma) (QC metrics prior to filtering cells shown in Figures S1A and S1B). After filtering very low expressed genes based on mean expression, we observed 12,756 and 11,395 genes across all time points in Ishikawa and T-47D cells, respectively. We first set out to identify determinants of mean expression levels by focusing on the 0-h time point (no E2 treatment). Genome-wide data, mostly on protein-DNA interactions, from publicly available sources[1,55,56] and experiments conducted for this study (Table S3) were quantified at promoter and enhancer regions. Due to variations in enhancer number and strength across genes, an aggregate enhancer score was used to capture the combined action of multiple enhancers (see STAR Methods) (Figure S1C). Genomic features were ranked by importance for classifying low (bottom 20% of genes), medium (middle 60%), and high (top 20% of genes) expression levels using the Boruta algorithm for feature selection,[57] which has been previously used to uncover determinants of expression noise in *Drosophila*.[58] For feature ranking, we grouped genes from both cell types to find mutual predictors, with the expectation that there are common underlying mechanisms for transcription control.

Elements of the pre-initiation complex and H3K27ac at the promoter ranked as the most important predictors for transcript levels (Figure 1A left). These features had stronger promoter signals at higher expressed genes (Figures 1B–1E), in agreement with previous literature.[59,60] Important factors at promoters and enhancers showed a general trend of increased signal for highly expressed genes (Figure 1A, right). Our dataset is strongly biased toward activating TFs and histone modifications found at active regulatory regions. Plotting the average promoter intensity compared with the average enhancer score across all confirmed datasets verifies that strong promoters and active enhancers are associated with higher gene expression levels (Figure 1F). Overall, the Boruta approach was successful at identifying known predictors of transcript levels.

Analysis of temporal trajectories indicates features that control estrogen response timing

We next analyzed genomic predictors of response timing using the scRNA-seq data following 0, 2, 4, and 8 h of estrogen treatment. Using dimensionality-reduction UMAP plots, the temporal progression of the E2 response in single cells can be observed, although cells are not fully separated by time point (Figures S1D and S1E). The clustering of cells suggests that the transcriptional response does not follow a tight temporal pattern and that variation in the E2 response exists between cells. In addition, T-47D cells exhibit distinct clusters prior to E2 treatment that do not appear to be cell cycle related based on *TOP2A* expression (Figure S1F). Based on a Wilcoxon rank-sum test,[61] there were between 491 and 1,146 differentially expressed genes for each cell type between any E2 treatment time point and the 0-h control (Figure S2A). scRNA-seq summed counts showed high concordance with previously published bulk RNA-seq data in the same cell lines (Figure S2B and S1C).[62] Compared with bulk RNA-seq, there are more differentially expressed genes with high expression (Figures S2B and S2C) and lower fold changes (Figures S2D and S2E), likely due to the increased statistical

CellPress
OPEN ACCESS

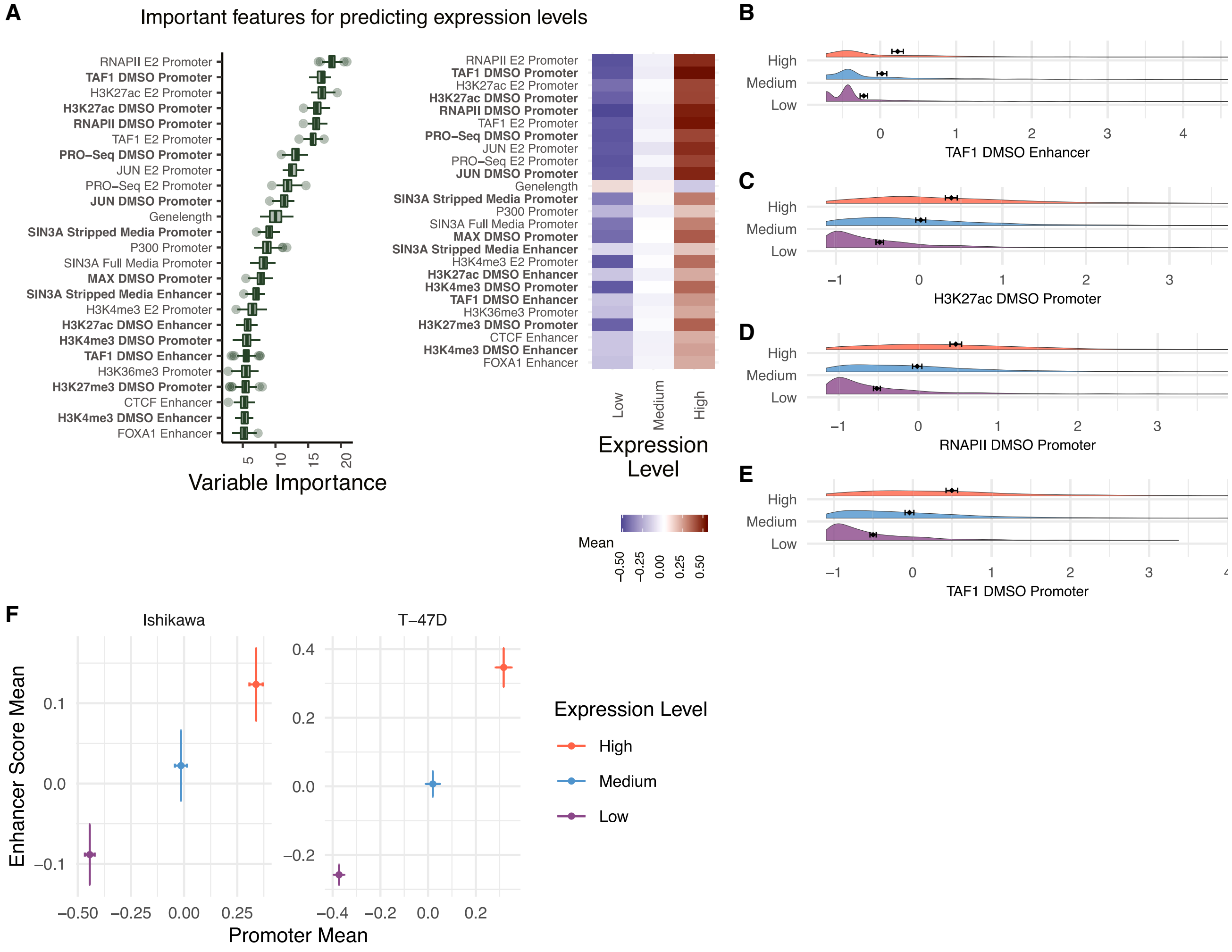

Figure 1. Several genomic features associate with gene expression levels

(A) (Left) Boruta feature ranking of genomic features shows importance of a feature for predicting mean levels. (Right) Average signal intensity for each genomic dataset, grouped by mean expression levels, is shown. Datasets shown in bold were performed in the absence of ER activation.

(B–E) Distributions of the top four most important ranked features in the absence of ER activation, separated by mean expression levels, show higher signal for High expression groups. The x axis represents *Z* scores and error bars show the mean ±95% confidence intervals.

(F) Mean enhancer score signal for all Boruta confirmed features vs. mean promoter signal across all confirmed features is shown where error bars show the mean ±95% confidence intervals. Axes do not represent the full range.

power of scRNA-seq for calling differential expression of highly expressed genes. However, there was still high overlap between differentially expressed genes at the matching 0-h vs. 8-h comparison. Of genes that occur in both single-cell and bulk datasets 48.1% and 73.6% of 8-h bulk RNA-seq genes overlap with 8-h scRNA-seq genes ($p < 2.2 \times 10^{-16}$, odds ratio = 6.11; and $p < 2.2 \times 10^{-16}$, odds ratio = 12.61; Fisher's exact test in Ishikawa and T-47D, respectively). In addition to the comparison with previously published bulk RNA-seq from 0- and 8-h E2 treatment, we performed bulk RNA-seq using the same time course as the scRNA-seq experiment. We observed highly significant overlaps between expression trajectories in both cell lines (Figure S2F). scRNA-seq can therefore be a valuable tool to capture subtle changes in gene expression following E2 treatment.

Based on the time point at which a gene is differentially regulated, genes were classified into temporal response trajectories for up- and down-regulated genes. One class of genes rapidly changes expression in response to E2 (termed Early genes), while another class changes more gradually and takes longer to reach a maximum response (termed Late genes). A representative set of control genes was also randomly selected using stratified sampling to mirror mean levels found in differential genes. Early-responding genes have a significant initial response to E2 by 2 h, then return toward baseline for both up- and down-regulation, consistent with previous reports of pulse-like expression in immediate-early genes[63] (Figures 2A and 2B). In contrast, genes classified as Late show a slow and steady response over time (Figures 2A and 2B). We found that both Early Up and Late Up genes were significantly enriched for the previously

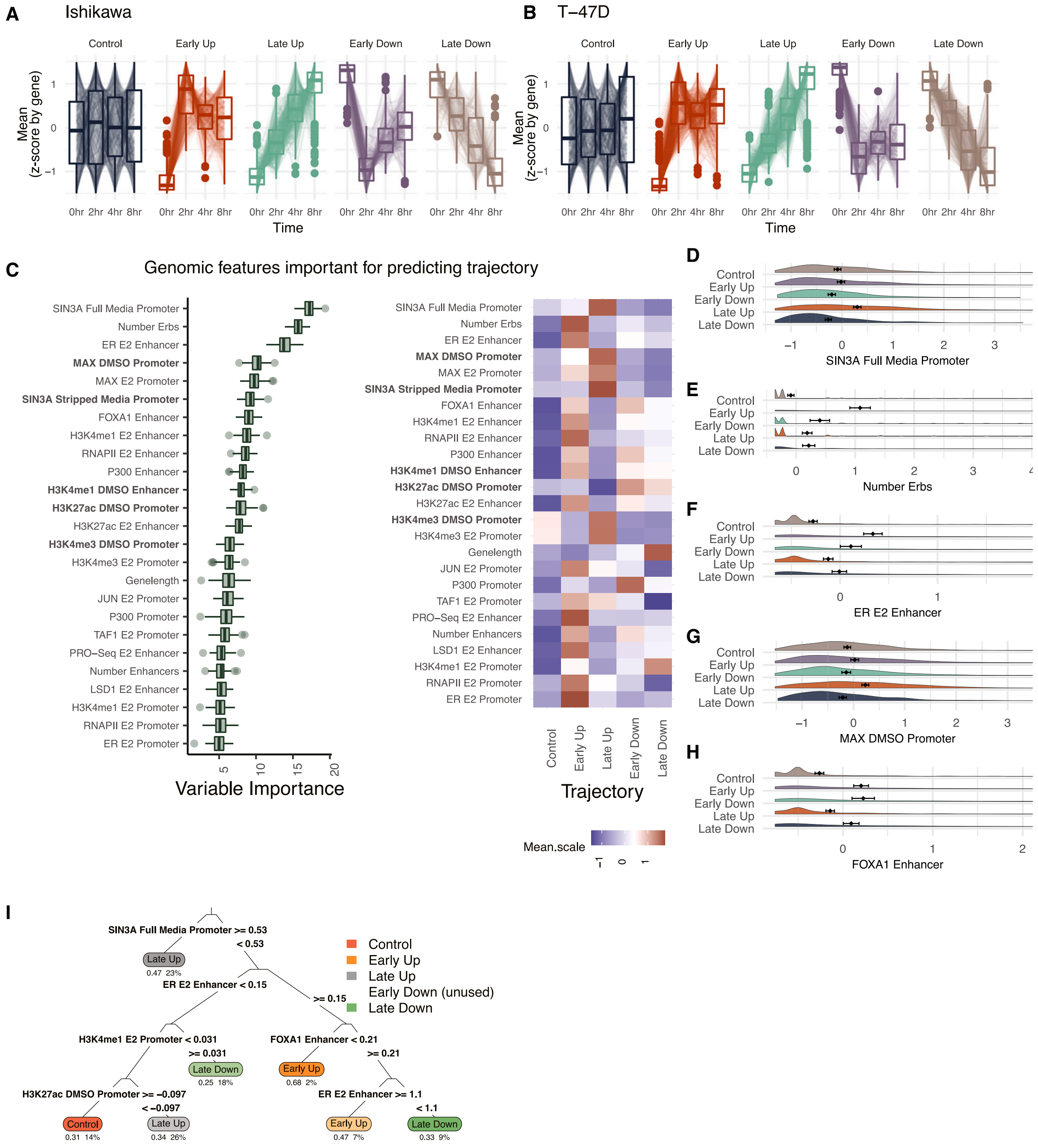

Figure 2. SIN3A and multiple ER-bound sites are the strongest predictors of transcriptional response timing

(A and B) *Z* scores for each gene across four time points are shown within different gene expression trajectories in (A) Ishikawa and (B) T-47D cells.

(C) (Left) Based on Boruta ranking, the top 25 most important features are shown for classifying gene trajectories. (Right) Heatmap displays the average signal by trajectory for each predictor. Datasets shown in bold were performed in the absence of ER activation.

(D–H) Distribution of signal (*Z* score) of the most important features for predicting temporal trajectories is shown.

(I) Decision tree shows the hierarchy of classification for predicting gene expression trajectories. The top four layers are shown.

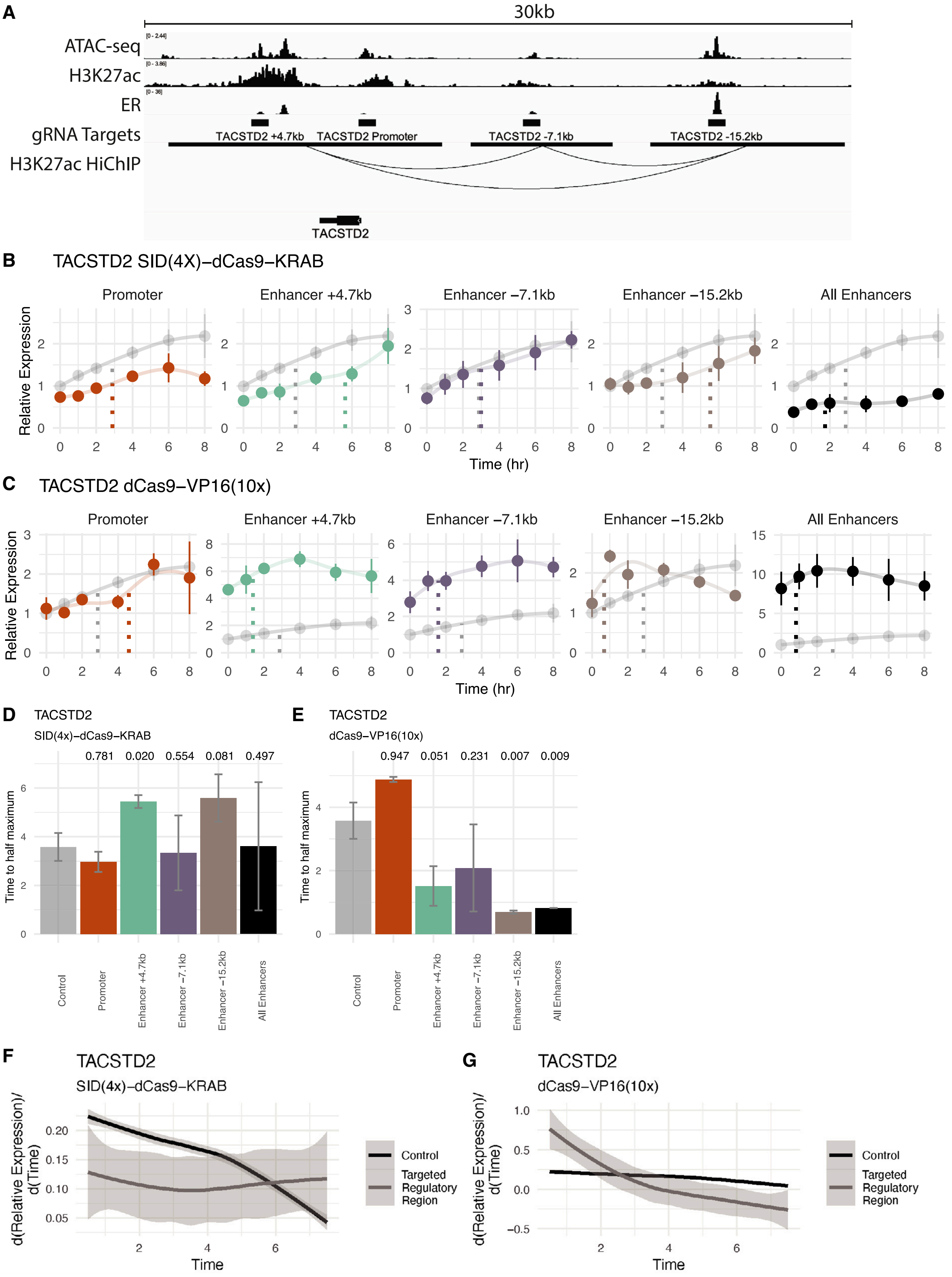

(legend on next page)

described hallmarks of estrogen response early and late (Figures S3A–S3D). As we have reported previously,[62,64] the transcriptional response to E2 is highly cell-type specific. However, genes with Up trajectories are more conserved between cell types than genes with Down trajectories (Figure S3E) ($p = 0.013$; two-sided t test).

The Boruta algorithm was used to identify predictors of temporal trajectories. SIN3A signal at the promoter was most predictive of gene expression trajectory and is associated with Late Up genes (Figures 2C and 2D). MAX, which is known to repress genes through recruitment of SIN3A,[65] was also classified as important and enriched at promoters of Late Up genes (Figure 2G). The number of ER binding sites (ERBS) that loop to the promoter and the ER signal at enhancers were the next most important features (Figure 2C). These two features are enriched in Early Up genes (Figures 2E and 2F). A higher number of total enhancers is enriched for genes that respond Early (Figure S3F); however, specific proteins (e.g., FOXA1) (Figure 2H) are more balanced between Early Up and Early Down genes than other factors (e.g., ER) that show preferential binding near Early Up genes. Together, these results suggest that the number of enhancers plays a prominent role in the temporal response of genes, but TFs at these sites, such as ER, help control the direction and timing of gene expression changes. An example of an optimal decision tree was computed to examine a potential hierarchy of factors determining a gene's temporal response (Figure 2I). This decision tree shows how SIN3A is the primary separator of genes into the Late Up trajectory, which may take precedence over ER signal. This analysis was run on the combined Ishikawa and T-47D data. When we perform the same analysis separately on each cell line, we find that the importance scores are highly correlated ($r = 0.69$; Figure S3G), indicating that shared mechanisms control timing across cell types. When analyzing promoter and enhancer activity separately, we found that promoter signal is highly correlated between cell types regardless of whether or not a gene exhibited the same response to E2 ($r = 0.74$ vs. 0.7). However, enhancer signal is less correlated between cell types and lower when a gene's trajectory is not conserved between cell types ($r = 0.39$ vs. 0.3), indicating that enhancer features play a larger role in cell-type-specific gene regulation by estrogen. Overall, Boruta analysis of temporal trajectories uncovers unique factors that may regulate transcription response timing and shows the association of multiple ER-bound enhancers with a rapid up-regulation in response to E2.

One potential confounding factor of the trajectory analysis is the impact of mRNA half-life, since the scRNA-seq measurements represent poly-adenylated mRNAs and not simply nascent transcripts. To evaluate how mRNA turnover could play a role in trajectory, we compared previously published mRNA half-life measurements[66] across E2 regulated genes (Figure S3H). We found that Early Down genes exhibited significantly shorter mRNA half-lives than other genes ($p < 2.2 \times 10^{-16}$, two-sided Kolmogorov-Smirnov test; average of 0.55 standard deviations below control genes). This finding suggests that fast mRNA turnover is important for quick down-regulation in response to estrogen treatment. To a lesser extent, we see that Early Up genes are also characterized by shorter half-lives ($p = 8.x10^{-12}$, two-sided Kolmogorov-Smirnov test; average of 0.29 standard deviations below control genes). This feature may help to explain the pattern of Early Up genes rapidly increasing by 2 h, but leveling off during the rest of the 8-h E2 treatment.

Functional perturbation of CREs alters temporal responses

To test the functional relationship between CREs and transcriptional response timing, dCas9-based activators and repressors were used to modulate the genomic activity of regulatory regions in Ishikawa cells, as T-47D cells exhibit low transfection efficiencies. Gene expression responses were then measured during a time course of E2 treatment using quantitative PCR. An SID(4x)-dCas9-KRAB construct was used for repression.[67] This construct can directly recruit SIN3A, a good predictor of Late gene expression responses. It also recruits histone deacetylases (HDACs),[68] corresponding to the low H3K27ac seen at Late Up genes. For activation, dCas9-VP16(10x) was used. We have previously shown that dCas9-VP16(10x) modulates expression from enhancers and induces acetylation at targeted regions.[69] dCas9-VP16(10x) recruits many activating cofactors, including members of the pre-initiation complex and p300, which are associated with Early genes.

TACSTD2 is an E2 regulated gene that is a prognostic indicator for endometrial cancer disease-free survival,[70] is overexpressed in some breast cancers,[71] and exhibits an Early Up trajectory. Targeting the enhancers of *TACSTD2*, marked by H3K27ac and ER binding (Figure 3A) with SID(4X)-dCas9-KRAB resulted in a slower, more gradual response to E2 when targeting two out of three individual enhancers (Figure 3B). Synthetic activation of the same *TACSTD2*-linked CREs led to a more rapid response when targeting most individual enhancers and the combination of all enhancers (Figure 3C). For enhancer repression, targeting enhancer +4.7 kb and enhancer −15.2 kb resulted in longer activation times compared with non-targeting controls based on time to half maximal expression (Figure 3D).

Figure 3. Functional manipulation of enhancer activity alters *TACSTD2* E2 response timing

(A) Chromatin immunoprecipitation (ChIP)-seq, assay for transposase-accessible chromatin with sequencing (ATAC-seq), and HiChIP genome browser tracks in Ishikawa cells show targeted regulatory regions surrounding *TACSTD2*.

(B and C) Expression trajectory of *TACSTD2* is displayed after E2 induction in Ishikawa cells following SID(4x)-dCas9-KRAB inhibition (B) or dCas9-VP16(10x) activation (C) targeted to regulatory regions. Error bars represent SEM ($n = 2$) and expression is relative to the 0-h time point in the control, which is from cells with *IL1RN* promoter targeting. Dotted lines show the time at half maximum for a given trajectory.

(D and E) Bar plot shows time to half maximal expression for each targeted regulatory region. Error bars represent SEM and p values (one-sided t test) are reported above each bar.

(F and G) Aggregate differential of loess regressions from (B) and (C) for all regulatory regions targeted (gray) by SID(4x)-dCas9-KRAB (F) or dCas9-VP16(10x) (G) compared with control (black). Shaded region represents 95% confidence interval.

Enhancer +4.7 kb and enhancer −15.2 kb exhibited slower activation rates, but reached similar activation levels at 8 h compared with controls, indicating that these enhancers can regulate the timing of a response without affecting overall levels. For enhancer activation, targeting most enhancers led to a decreased time to half maximal expression (Figure 3E). Again, we see that enhancer −15.2 kb changes expression timing without affecting overall transcript abundance. Analysis of the slopes revealed a generally slower initial activation when *TACSTD2* regulatory regions were inhibited (Figure 3F) and generally increased initial slopes when *TACSTD2* regulatory regions were activated (Figure 3G). These results imply that decreasing enhancer activity can reduce initial activation rates, while activating enhancers can potentiate quicker responses to E2.

When targeting five putative enhancers as well as the promoter of *TGFA*, an Early Up gene, with SID(4X)-dCas9-KRAB, we again observed a more gradual expression response to E2 (Figure S4A). We also sought to speed up a Late gene by targeting dCas9-VP16(10x) to the enhancers and the promoter of *PEG10*. We observed an overall faster response when targeting PEG10's regulatory elements (Figure S4B). The most substantial effects on *TGFA*, in terms of time to half maximal expression, were observed when targeting enhancer −43 kb, enhancer −62 kb, or all enhancers simultaneously (Figure S4C). For *PEG10*, baseline levels were increased by dCas9-VP16(10x) targeting at all CREs, which resulted in more variability in the timing analysis (Figure S4D). Analysis of slopes showed that inhibition of *TGFA* regulatory regions slowed activation rates between early time points, followed by increased rates from 6 to 8 h relative to the control trajectory (Figure S4E). These results are consistent with our *TACSTD2* findings and indicate that decreasing enhancer activity slows the transcriptional response. Analysis of slopes for *PEG10* showed that activation of *PEG10* enhancers led to earlier responses to E2, which later converge with control rates (Figure S4F). Overall, at these three genes, the activity of enhancers controls the E2 response trajectory.

An enhancer-promoter dichotomy controls gene expression noise

Genes were separated into three levels of variation to find determinants of expression noise at the 0-h time point. In scRNA-seq, low gene expression levels often have high noise due to the dropout effects of capturing RNAs from the limited amount of RNA in a single-cell and technical variation in scRNA-seq is related to mean levels.[72] To examine mean-independent noise, we used an adjusted coefficient of variation (CV), which is calculated as the residuals of a generalized additive model (GAM) where CV is fit to the mean (Figures S5A–S5D, see STAR Methods). To remove any leftover mean effects, genes were labeled as high or low noise based on whether they were in the top 20% or bottom 20% of adjusted CV for 10 different mean bins from the 0-h time point of both cell types.

The strongest predictors of noise levels were promoter signals of JUN, SIN3A, and tri-methylation at histone H3 lysine 4 (H3K4me3), each associated with low noise (Figures 4A–4D). Generally, a strong promoter signal was related to low noise across features, with some exceptions, such as p300 (Figure 4A, right panel). Most enhancer features were associated with high noise, with ER and FOXA1 at enhancers being the most predictive (Figure 4A). The strong association between H3K4me3 and low amounts of noise supports a previously reported correlation between H3K4me3 breadth and transcriptional consistency.[73] The above analysis was performed on the combined Ishikawa and T-47D data; however, the importance scores of features when run separately on the cell types were correlated (r = 0.58; Figure S5E), indicating that common features control transcriptional noise.

These results motivated the broader evaluation of how noise relates to promoter and enhancer activity. Analysis of the average promoter intensity across all confirmed datasets and the average enhancer score revealed an inverse relationship between promoters and enhancers (Figures 4E and S6A). Genes with high noise levels had high enhancer scores and low promoter signals. Conversely, genes with low noise levels had low enhancer scores and high promoter signals. To confirm this relationship in a third cell type, we analyzed publicly available scRNA-seq and genomic data from LNCaP cells, a prostate cancer cell line. The same association was observed between enhancer-driven gene regulation and higher noise (Figure S6B). Consistent with these findings, more enhancers connected to a gene associate with greater noise (Figures 4F and 4G). These results indicate that enhancer-driven transcription regulation is less consistent across individual cells than promoter-driven transcription regulation.

Shared features of expression levels, timing, and noise

Expression levels, noise, and timing analysis uncovered different importance rankings for genomic features. Importance scores fell roughly into five common patterns across our three analysis types (Figure 5A). The largest three patterns consisted of features whose importance scores were highly enriched for a single analysis. Two smaller patterns were composed of features important for both mean and noise or both trajectory and noise. In general, the importance scores from the Boruta algorithm are more similar between noise and mean or noise and trajectory compared with mean and trajectory, as seen from the first two PCA dimensions calculated from the feature importance matrix (Figure 5B) and the correlation between importance scores (Figures S6C–S6E). The relationship between noise and our other analyses suggests that noise may be an intermediary between baseline levels and temporal regulation and that mean levels do not strongly influence response trajectory. Generally, we see that most features specifically associated with mean levels are found at promoters, while noise and trajectory utilize promoter and enhancer features more evenly (Figure 5A). Enhancer features important for predicting mean levels are also likely to predict noise. Together, these results indicate that enhancers are more critical for noise and trajectory and that many genomic signals preferentially predict levels, noise, or trajectory.

Since Boruta importance scores do not capture directionality, we examined the group with the maximum signal for each feature (Figure S6F). Promoter features are generally associated with high mean levels and low noise. Enhancer features are also associated with increased mean levels, but contrary to

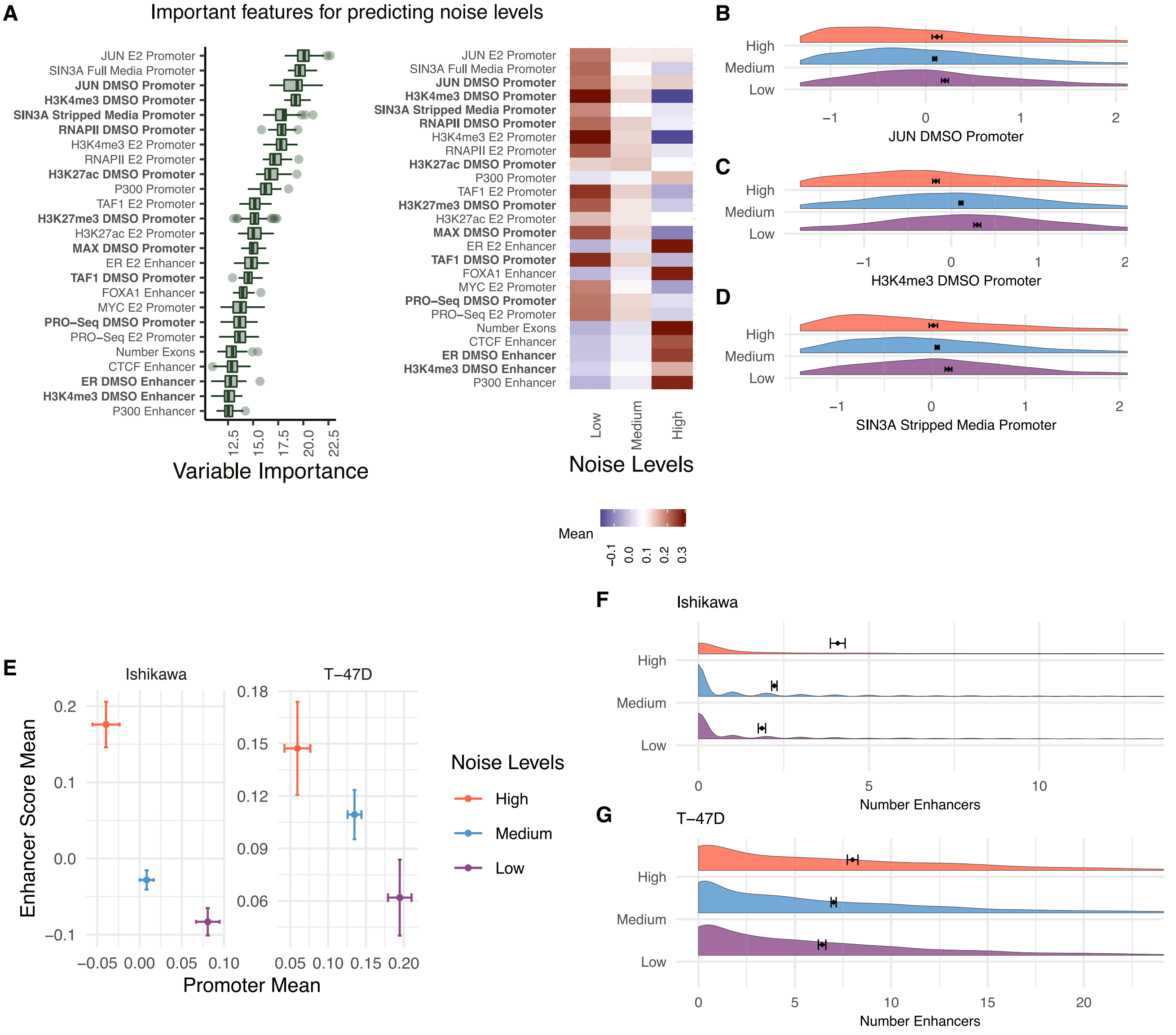

Figure 4. Features associated with noise levels indicate a balance between active promoters and active enhancers

(A) (Left) Boruta feature rankings shows features predictive of noise levels at the 0-h time point. (Right) Average signal intensity is shown by noise group for top ranked features. Datasets shown in bold were performed in the absence of ER activation.

(B–D) Distribution of signal for top ranked noise-predicting features in the absence of ER activation are shown with *Z* scores on the x axis.

(E) Mean enhancer signal score for all Boruta confirmed features vs. mean promoter signal across all confirmed features for each noise level exhibits an inverse relationship. Error bars show 95% confidence intervals and axes do not represent the full range; full distribution shown in Figure S6A.

(F and G) Distribution of enhancer counts per gene, separated by noise level, are shown for (F) Ishikawa and (G) T-47D cells.

promoters, they show an association with high noise levels. Enhancer scores are almost always the highest for Early Up trajectories. As high enhancer scores are associated with Early Up trajectories and high noise, we performed a gene level comparison of noise at the 0-h time point and trajectory classification. We found that genes that respond quickly to E2 treatment are more likely to exhibit high noise at the 0-h time point (Figure 5C). Our results suggest that active enhancers drive high noise and rapid up-regulation in response to E2, while promoters consistently drive low noise and high mean expression.

Co-expression of genes is associated with looping, timing, and noise levels

scRNA-seq offers a unique advantage in studying the co-regulation of genes on a cell-by-cell basis and the possible mechanisms that underlie co-regulation. Using the H3K27ac HiChIP data, we found that looping can affect co-expression in several ways. First, we found that genes whose promoters loop together correlate significantly more than groups of randomly paired control genes at the 0-h time point (Figures 6A and 6B). Genes whose promoters both loop to a shared enhancer are

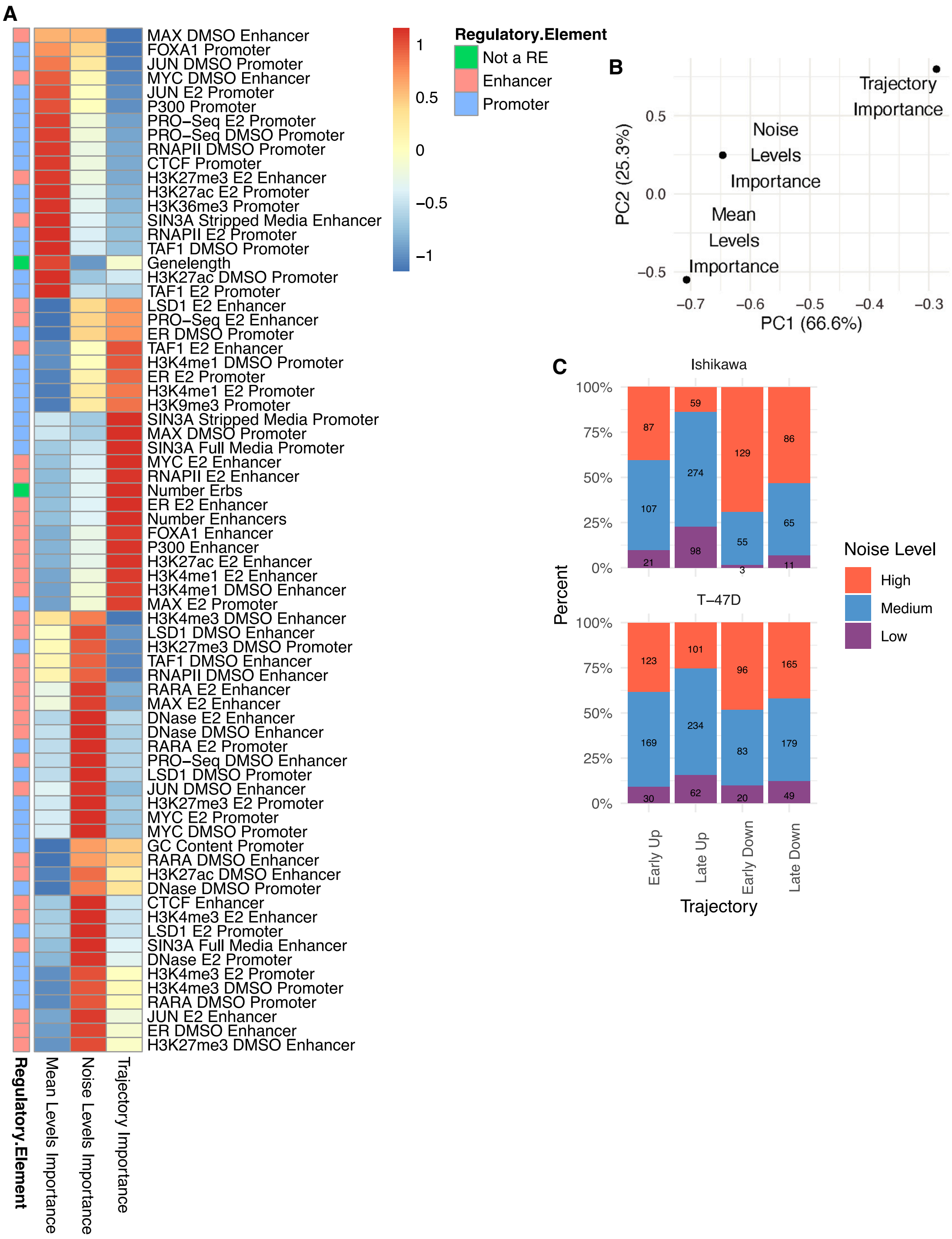

Figure 5. Importance comparison shows that mean and trajectory are regulated by distinct genomic features

(A) Heatmap shows importance scores from each analysis type, normalized by column, and scaled by row.

(B) PCA plot based on importance scores shows the relationship of importance scores for mean levels, noise, and trajectory. Percentages denote percent of variance explained by each principal component.

(C) Bar plot shows the percentage of genes for each trajectory that are classified into each noise classification. Numbers on bars refer to counts of genes.

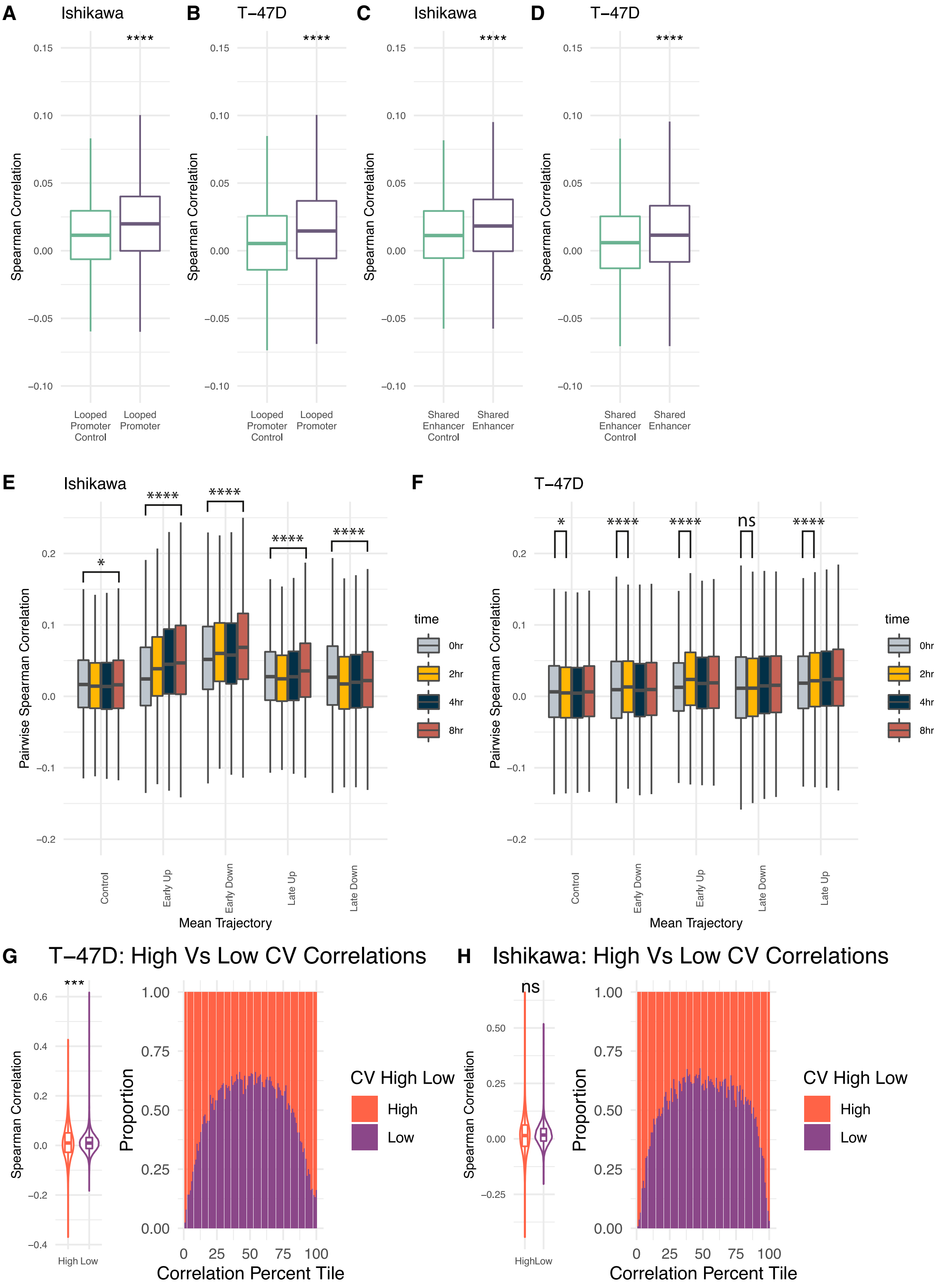

Figure 6. Co-expression levels track with looping, trajectory, and levels of noise

(A and B) Pairs of genes with promoters that loop to one another are significantly more correlated across cells at the 0-h time point than randomly selected gene pairs for Ishikawa (A) and T-47D (B).

(C and D) Pairs of genes with a shared enhancer are more correlated than randomly paired genes for Ishikawa (C) and T-47D (D).

(legend continued on next page)

significantly more correlated across single cells (Figures 6C and 6D). These results indicate that the 3D genome structure may be involved or associated with gene co-expression across single cells.

We next evaluated co-expression during the E2 treatment time course. Co-expression was measured using pairwise Spearman correlation in single cells. In Ishikawa cells, both Early Up and Early Down genes show increasing pairwise co-expression over time (Figure 6E). Genes that respond late exhibited less change in correlation, with Late Up genes increasing correlation slightly by 8 h and Late Down genes slightly decreasing in correlation. In T-47D cells, we see the most significant increase in co-correlation at 2 h for Early Up and Early Down genes (Figure 6F). Late Up and Late Down genes show slight increases in correlation during the time course.

The levels of noise also change the probability of two genes being correlated. Perhaps expected, genes with high noise levels also show a broader distribution in their pairwise correlations, resulting in genes with high noise being more likely to have extremely correlated or anticorrelated expression with each other than low noise genes (Figures 6G and 6H). These results indicate that 3D interactions, control of temporal trajectory, and noise regulation can impact gene co-regulation.

DISCUSSION

To investigate the genomic underpinnings of the temporal transcriptional response to estrogen, we conducted scRNA-seq at several time points in two human cell lines. scRNA-seq was able to capture more subtle changes in gene expression of highly expressed genes compared with bulk RNA-seq due to the increased statistical power. Using a feature ranking approach, we identified several features associated with E2 response timing, including more ER-bound enhancers regulating Early Up genes and SIN3A signal at the promoter of Late Up genes. In general, multiple enhancers are more predictive of Early gene trajectories. Functional evaluation of enhancers revealed that multiple enhancers regulate timing at each gene tested as activation and repression of several enhancers causes changes in a gene's temporal response to estrogen. From these studies, we conclude that an active enhancer repertoire is important for rapid gene responses to estrogen. Active enhancers may present chromatin that is more open to ER binding. Alternatively, other TFs already present at enhancers could stabilize the binding of ER, permitting ER to activate gene expression immediately. Contrastingly, SIN3A and MAX at the promoter, known to repress gene expression together,[65] slow a gene's response to E2, even when a gene is associated with strong ER-bound enhancers. Activation of gene expression may first require removing repressive signals at the promoter, explaining the more gradual responses. A similar mechanism has been described in an enhancer context, where inactive enhancers must first be activated by TFs before activation of gene expression can occur, causing more gradual gene expression changes.[25] While transcriptional responses to estrogen,[64] as well as underlying gene regulatory elements,[62] are highly cell-type specific, we found that the genomic features associated with the transcriptional response are shared between cell types, indicating that common mechanisms drive how genes respond to estrogen stimulation.

One unexpected and exciting finding from our analysis is that enhancer and promoter activities relate to expression noise in opposing manners. Active promoters are associated with low noise levels, whereas multiple active enhancers are associated with high noise levels. In concordance with this observation, synthetic activation of promoters drives lower noise levels at several genes.[74] Additionally, activation of multiple enhancers causes high noise at the NF-κB locus.[46] Our results support a unified model where a balance between enhancers and promoters controls noise. Both intrinsic and extrinsic noise could potentially explain the observed noise distributions.[75] If intrinsic noise is the driving factor, we expect promoters to cause high-frequency, near-constant transcription levels and enhancers to cause infrequent, high-amplitude bursts of expression.[8] Noise caused by CREs could also be due to extrinsic noise. Promoters may lead to low noise, as fluctuations in upstream factors may be insignificant compared with activation by an ensemble of TFs bound to the promoter. In contrast, enhancers may drive higher noise levels by increasing sensitivity to upstream factors, which is consistent with our observation that enhancers drive rapid temporal responses to estrogen.

For a gene to respond quickly to a signal, it must be sensitized to incoming signals, which may inherently drive higher levels of noise. A noise-robustness tradeoff has been proposed previously when observing changes in gene expression over developmental time in *Drosophila*[58] and in a mathematical framework that showed variation is necessary for a gene's responsiveness.[76] However, a regulatory mechanism has not been found. Our results point to multiple enhancers being a primary genomic feature associated with both high expression noise and rapid response timing. Additionally, we found that a strong promoter is likely to cause more robust gene expression but limited responsiveness. While we identified these patterns across the full dataset, a small set of individual genes can respond quickly to a signal without high cell-cell variation (Figure 5C) and the question remains as to how these genes maintain low noise and fast response times. We also found that genes that are quickly down-regulated by estrogen are more likely to exhibit higher noise than genes that are quickly up-regulated by estrogen. This pattern could be due to genes that are primarily enhancer driven having higher noise and being more susceptible to down-regulation, potentially through enhancer rewiring because of ER activity and competition for factors that are critical for enhancer activity.

(E and F) Distribution of pairwise Spearman correlation for genes within different trajectories is shown for Ishikawa (E) and T-47D (F).
(G and H) Range of pairwise correlations for high noise levels is greater than the range for pairs of low noise genes in T-47D (G) and Ishikawa (H). (Left) Distribution of Spearman pairwise correlations for genes with high and low noise. (Right) Spearman correlations were grouped into quantiles and bars show proportion at each quantile that are pairs of low or high noise genes. Significance values for all subpanels are as follows (based on Bonferroni corrected two-sided Wilcoxon tests): $*p < 0.05$; $**p < 1 \times 10^{-5}$; $***p < 1 \times 10^{-10}$; $****p < 1 \times 10^{-15}$.

Co-expression analysis of gene pairs showed that co-expression properties depend on looping, timing, and noise. We found that genes with shared enhancers and looped promoters correlate more in individual cells, genes with different trajectories correlate differently over time, and genes with high noise levels are more likely to be strongly correlated or anticorrelated. While co-expression correlation effects are modest overall and it is unclear what levels of co-expression are biologically meaningful, these observations could have implications for gene regulatory networks (GRNs) in single cells, as co-expression often underlies regulatory networks. Dynamic adjustment of regulatory networks may have critical functional outcomes for a cell population.[77] For example, GRNs that confer resistance to therapeutics may occur at distinct time points following treatment.[78] Our results indicate that genes with high noise may lead to a broader range of implemented regulatory networks across single cells, enhancing cellular heterogeneity. Further studies into functional GRNs are warranted to determine how noise and timing affect single-cell phenotypes through the co-expression of many genes. Overall, our study shows that enhancers and promoters can play distinct roles in the timing and variation of a transcriptional response.

Limitations of the study

It is important to note that we analyzed total poly-adenylated RNA in this study. Since mRNA degradation can happen on different time scales across genes,[79] some genes may be misclassified in their transcriptional response to estrogen. In fact, we found that Early Down genes had shorter mRNA half-lives in previously published datasets. This observation is consistent with quickly down-regulated genes requiring fast mRNA turnover to be observed from poly-adenylated RNA, since overall transcript levels would need to be significantly reduced in a short time period. The confounding effect of mRNA stability on down-regulated genes may partially explain why we did not find features that consistently associate with Early/Late Down genes. The lack of strong association between the genomic features we studied and down-regulated genes could also be related to a focus on features related to gene activation and a stronger connection between ER and gene activation as opposed to repression.[67] Variation in mRNA half-lives could also contribute to the amount of noise measured in this study, which would explain the observation that Early Down genes exhibit higher noise and shorter mRNA half-lives. Single-cell measurements of either nascent transcription or mRNA decay would be needed to deconvolute the effects of the mRNA life cycle on transcription timing and noise; however, this type of analysis would be challenging with current technologies.

STAR★METHODS

Detailed methods are provided in the online version of this paper and include the following:

- KEY RESOURCES TABLE
- RESOURCES AVAILABILITY
  - Lead contact
  - Materials availability
  - Data and code availability
- EXPERIMENTAL MODEL AND SUBJECT DETAILS
  - Cell lines
- METHOD DETAILS
  - ChIP-seq
  - H3K27ac HiChIP
  - PRO-seq
  - scRNA-seq
  - scRNA-seq analysis: Classification of trajectory and noise levels
  - Feature importance analysis
  - Generation of stable dCas9-VP16(10x) cell lines
  - gRNA design and transfection
  - RNA isolation and qPCR gene expression analysis
  - Bulk RNA-seq experiments and analysis
  - Co-expression analysis
- QUANTIFICATION AND STATISTICAL ANALYSIS

SUPPLEMENTAL INFORMATION

Supplemental information can be found online at https://doi.org/10.1016/j.xgen.2024.100542.

ACKNOWLEDGMENTS

Funding for this work came from the National Institutes of Health (NIH)/National Human Genome Research Institute (NHGRI) R01 HG008974 to J.G. and the Huntsman Cancer Institute. Research reported in this publication utilized the High-Throughput Genomics Shared Resource at the University of Utah and was supported by NIH/National Cancer Institute (NCI) award P30 CA042014. We thank Craig M. Rush for experimental guidance, Jeffery Vahrenkamp for analysis advice, and Gertz lab members for their suggestions on the study and manuscript.

AUTHOR CONTRIBUTIONS

Conceptualization, M.G.-H. and J.G.; methodology, M.G.-H. and J.G.; investigation, M.G.-H., H.A., N.K., K.L.M., and E.M.W; formal analysis, M.G.-H., J.G., A.R., and X.Z.; writing - original draft, M.G.-H. and J.G.; writing – review & editing, all authors; supervision, J.G., J.L., and X.Z.; funding acquisition, J.G.

DECLARATION OF INTERESTS

The authors declare no competing interests.

Received: March 16, 2023
Revised: October 26, 2023
Accepted: March 27, 2024
Published: April 24, 2024

REFERENCES

1. ENCODE Project Consortium (2012). An integrated encyclopedia of DNA elements in the human genome. Nature *489*, 57–74. https://doi.org/10.1038/nature11247.
2. Roadmap Epigenomics Consortium; Kundaje, A., Meuleman, W., Ernst, J., Bilenky, M., Yen, A., Heravi-Moussavi, A., Kheradpour, P., Zhang, Z., Wang, J., et al. (2015). Integrative analysis of 111 reference human epigenomes. Nature *518*, 317–330. https://doi.org/10.1038/nature14248.
3. Zhang, J., Lee, D., Dhiman, V., Jiang, P., Xu, J., McGillivray, P., Yang, H., Liu, J., Meyerson, W., Clarke, D., et al. (2020). An integrative ENCODE resource for cancer genomics. Nat. Commun. *11*, 3696. https://doi.org/10.1038/s41467-020-14743-w.
4. MacKenzie, A., Hing, B., and Davidson, S. (2013). Exploring the effects of polymorphisms on cis-regulatory signal transduction response. Trends Mol. Med. *19*, 99–107. https://doi.org/10.1016/j.molmed.2012.11.003.
5. Wei, Y., Gokhale, R.H., Sonnenschein, A., Montgomery, K.M., Ingersoll, A., and Arnosti, D.N. (2016). Complex cis-regulatory landscape of the

insulin receptor gene underlies the broad expression of a central signaling regulator. Development *143*, 3591–3603.

6. Kolch, W., Halasz, M., Granovskaya, M., and Kholodenko, B.N. (2015). The dynamic control of signal transduction networks in cancer cells. Nat. Rev. Cancer *15*, 515–527. https://doi.org/10.1038/nrc3983.
7. Raser, J.M., and O'Shea, E.K. (2005). Noise in Gene Expression: Origins, Consequences, and Control. Science *309*, 2010–2013. https://doi.org/10.1126/science.1105891.
8. Raj, A., and Van Oudenaarden, A. (2008). Nature, Nurture, or Chance: Stochastic Gene Expression and Its Consequences. Cell *135*, 216–226. https://doi.org/10.1016/j.cell.2008.09.050.
9. Chamberlain, C.E., Jeong, J., Guo, C., Allen, B.L., and McMahon, A.P. (2008). Notochord-derived Shh concentrates in close association with the apically positioned basal body in neural target cells and forms a dynamic gradient during neural patterning. Development *135*, 1097–1106. https://doi.org/10.1242/dev.013086.
10. Basma, H., Soto-Gutiérrez, A., Yannam, G.R., Liu, L., Ito, R., Yamamoto, T., Ellis, E., Carson, S.D., Sato, S., Chen, Y., et al. (2009). Differentiation and transplantation of human embryonic stem cell-derived hepatocytes. Gastroenterology *136*, 990–999. https://doi.org/10.1053/j.gastro.2008.10.047.
11. Konstantinides, N., Holguera, I., Rossi, A.M., Escobar, A., Dudragne, L., Chen, Y.-C., Tran, T.N., Martínez Jaimes, A.M., Özel, M.N., Simon, F., et al. (2022). A complete temporal transcription factor series in the fly visual system. Nature *604*, 316–322. https://doi.org/10.1038/s41586-022-04564-w.
12. Behar, M., and Hoffmann, A. (2010). Understanding the temporal codes of intra-cellular signals. Curr. Opin. Genet. Dev. *20*, 684–693. https://doi.org/10.1016/j.gde.2010.09.007.
13. Krakauer, D.C., Page, K.M., and Sealfon, S. (2002). Module dynamics of the GnRH signal transduction network. J. Theor. Biol. *218*, 457–470.
14. Uribe, M.L., Marrocco, I., and Yarden, Y. (2021). EGFR in cancer: Signaling mechanisms, drugs, and acquired resistance. Cancers *13*, 2748.
15. Sheng, M., and Greenberg, M.E. (1990). The regulation and function of c-fos and other immediate early genes in the nervous system. Neuron *4*, 477–485. https://doi.org/10.1016/0896-6273(90)90106-P.
16. Uhlitz, F., Sieber, A., Wyler, E., Fritsche-Guenther, R., Meisig, J., Landthaler, M., Klinger, B., and Blüthgen, N. (2017). An immediate–late gene expression module decodes ERK signal duration. Mol. Syst. Biol. *13*, 944.
17. Gandhi, S.J., Zenklusen, D., Lionnet, T., and Singer, R.H. (2011). Transcription of functionally related constitutive genes is not coordinated. Nat. Struct. Mol. Biol. *18*, 27–34. https://doi.org/10.1038/nsmb.1934.
18. Szustakowski, J.D., Kosinski, P.A., Marrese, C.A., Lee, J.-H., Elliman, S.J., Nirmala, N., and Kemp, D.M. (2007). Dynamic resolution of functionally related gene sets in response to acute heat stress. BMC Mol. Biol. *8*, 46. https://doi.org/10.1186/1471-2199-8-46.
19. Schnoes, K.K., Jaffe, I.Z., Iyer, L., Dabreo, A., Aronovitz, M., Newfell, B., Hansen, U., Rosano, G., and Mendelsohn, M.E. (2008). Research Resource: Rapid Recruitment of Temporally Distinct Vascular Gene Sets by Estrogen. Mol. Endocrinol. *22*, 2544–2556. https://doi.org/10.1210/me.2008-0044.
20. Tullai, J.W., Schaffer, M.E., Mullenbrock, S., Sholder, G., Kasif, S., and Cooper, G.M. (2007). Immediate-Early and Delayed Primary Response Genes Are Distinct in Function and Genomic Architecture. J. Biol. Chem. *282*, 23981–23995. https://doi.org/10.1074/jbc.m702044200.
21. Murai, J., Zhang, H., Pongor, L., Tang, S.-W., Jo, U., Moribe, F., Ma, Y., Tomita, M., and Pommier, Y. (2020). Chromatin Remodeling and Immediate Early Gene Activation by SLFN11 in Response to Replication Stress. Cell Rep. *30*, 4137–4151.e6. https://doi.org/10.1016/j.celrep.2020.02.117.
22. Simeonov, D.R., Gowen, B.G., Boontanrart, M., Roth, T.L., Gagnon, J.D., Mumbach, M.R., Satpathy, A.T., Lee, Y., Bray, N.L., Chan, A.Y., et al. (2017). Discovery of stimulation-responsive immune enhancers with CRISPR activation. Nature *549*, 111–115. https://doi.org/10.1038/nature23875.
23. Juan, A.H., and Ruddle, F.H. (2003). Enhancer timing of Hox gene expression: deletion of the endogenous Hoxc8 early enhancer. Development *130*, 4823–4834. https://doi.org/10.1242/dev.00672.
24. Hah, N., Benner, C., Chong, L.-W., Yu, R.T., Downes, M., and Evans, R.M. (2015). Inflammation-sensitive super enhancers form domains of coordinately regulated enhancer RNAs. Proc. Natl. Acad. Sci. USA *112*, E297–E302. https://doi.org/10.1073/pnas.1424028112.
25. Ostuni, R., Piccolo, V., Barozzi, I., Polletti, S., Termanini, A., Bonifacio, S., Curina, A., Prosperini, E., Ghisletti, S., and Natoli, G. (2013). Latent Enhancers Activated by Stimulation in Differentiated Cells. Cell *152*, 157–171. https://doi.org/10.1016/j.cell.2012.12.018.
26. Elowitz, M.B., Levine, A.J., Siggia, E.D., and Swain, P.S. (2002). Stochastic Gene Expression in a Single Cell. Science *297*, 1183–1186. https://doi.org/10.1126/science.1070919.
27. Fraser, L.C.R., Dikdan, R.J., Dey, S., Singh, A., and Tyagi, S. (2021). Reduction in gene expression noise by targeted increase in accessibility at gene loci. Proc. Natl. Acad. Sci. USA *118*, e2018640118. https://doi.org/10.1073/pnas.2018640118.
28. Pedraza, J.M., Garcia, D.A., and Pérez-Ortiz, M.F. (2018). Noise, information and fitness in changing environments. Front. Physiol. *6*, 83.
29. Wollman, R. (2018). Robustness, Accuracy, and Cell State Heterogeneity in Biological Systems. Curr. Opin. Struct. Biol. *8*, 46–50. https://doi.org/10.1016/j.coisb.2017.11.009.
30. Desai, R.V., Chen, X., Martin, B., Chaturvedi, S., Hwang, D.W., Li, W., Yu, C., Ding, S., Thomson, M., Singer, R.H., et al. (2021). A DNA repair pathway can regulate transcriptional noise to promote cell fate transitions. Science *373*, eabc6506. https://doi.org/10.1126/science.abc6506.
31. Suderman, R., Bachman, J.A., Smith, A., Sorger, P.K., and Deeds, E.J. (2017). Fundamental trade-offs between information flow in single cells and cellular populations. Proc. Natl. Acad. Sci. USA *114*, 5755–5760. https://doi.org/10.1073/pnas.1615660114.
32. Azpeitia, E., Balanzario, E.P., and Wagner, A. (2020). Signaling pathways have an inherent need for noise to acquire information. BMC Bioinf. *21*, 462. https://doi.org/10.1186/s12859-020-03778-x.
33. Han, R., Huang, G., Wang, Y., Xu, Y., Hu, Y., Jiang, W., Wang, T., Xiao, T., and Zheng, D. (2016). Increased gene expression noise in human cancers is correlated with low p53 and immune activities as well as late stage cancer. Oncotarget *7*, 72011–72020. https://doi.org/10.18632/oncotarget.12457.
34. Qin, S., Jiang, J., Lu, Y., Nice, E.C., Huang, C., Zhang, J., and He, W. (2020). Emerging role of tumor cell plasticity in modifying therapeutic response. Signal Transduct. Targeted Ther. *5*, 228. https://doi.org/10.1038/s41392-020-00313-5.
35. Shaffer, S.M., Dunagin, M.C., Torborg, S.R., Torre, E.A., Emert, B., Krepler, C., Beqiri, M., Sproesser, K., Brafford, P.A., Xiao, M., et al. (2017). Rare cell variability and drug-induced reprogramming as a mode of cancer drug resistance. Nature *546*, 431–435. https://doi.org/10.1038/nature22794.
36. Fidler, I.J. (1978). Tumor heterogeneity and the biology of cancer invasion and metastasis. Cancer Res. *38*, 2651–2660.
37. Nguyen, A., Yoshida, M., Goodarzi, H., and Tavazoie, S.F. (2016). Highly variable cancer subpopulations that exhibit enhanced transcriptome variability and metastatic fitness. Nat. Commun. *7*, 11246–11313.
38. Larsson, A.J.M., Johnsson, P., Hagemann-Jensen, M., Hartmanis, L., Faridani, O.R., Reinius, B., Segerstolpe, Å., Rivera, C.M., Ren, B., and Sandberg, R. (2019). Genomic encoding of transcriptional burst kinetics. Nature *565*, 251–254. https://doi.org/10.1038/s41586-018-0836-1.

39. Choi, J.K., and Kim, Y.-J. (2009). Intrinsic variability of gene expression encoded in nucleosome positioning sequences. Nat. Genet. *41*, 498–503. https://doi.org/10.1038/ng.319.

40. Dadiani, M., Van Dijk, D., Segal, B., Field, Y., Ben-Artzi, G., Raveh-Sadka, T., Levo, M., Kaplow, I., Weinberger, A., and Segal, E. (2013). Two DNA-encoded strategies for increasing expression with opposing effects on promoter dynamics and transcriptional noise. Genome Res. *23*, 966–976. https://doi.org/10.1101/gr.149096.112.

41. Nicolas, D., Zoller, B., Suter, D.M., and Naef, F. (2018). Modulation of transcriptional burst frequency by histone acetylation. Proc. Natl. Acad. Sci. USA *115*, 7153–7158. https://doi.org/10.1073/pnas.1722330115.

42. Wu, S., Li, K., Li, Y., Zhao, T., Li, T., Yang, Y.-F., and Qian, W. (2017). Independent regulation of gene expression level and noise by histone modifications. PLoS Comput. Biol. *13*, e1005585. https://doi.org/10.1371/journal.pcbi.1005585.

43. Urban, E.A., and Johnston, R.J., Jr. (2018). Buffering and Amplifying Transcriptional Noise During Cell Fate Specification. Front. Genet. *9*, 591. https://doi.org/10.3389/fgene.2018.00591.

44. Parab, L., Pal, S., and Dhar, R. (2022). Transcription factor binding process is the primary driver of noise in gene expression. PLoS Genet. *18*, e1010535.

45. Hnisz, D., Shrinivas, K., Young, R.A., Chakraborty, A.K., and Sharp, P.A. (2017). A Phase Separation Model for Transcriptional Control. Cell *169*, 13–23. https://doi.org/10.1016/j.cell.2017.02.007.

46. Wibisana, J.N., Inaba, T., Shinohara, H., Yumoto, N., Hayashi, T., Umeda, M., Ebisawa, M., Nikaido, I., Sako, Y., and Okada, M. (2022). Enhanced transcriptional heterogeneity mediated by NF-κB super-enhancers. PLoS Genet. *18*, e1010235. https://doi.org/10.1371/journal.pgen.1010235.

47. Björnström, L., and Sjöberg, M. (2005). Mechanisms of estrogen receptor signaling: convergence of genomic and nongenomic actions on target genes. Mol. Endocrinol. *19*, 833–842.

48. Rodriguez, A.C., Blanchard, Z., Maurer, K.A., and Gertz, J. (2019). Estrogen Signaling in Endometrial Cancer: a Key Oncogenic Pathway with Several Open Questions. Horm. Cancer *10*, 51–63. https://doi.org/10.1007/s12672-019-0358-9.

49. Stanford, J.L., Szklo, M., and Brinton, L.A. (1986). Estrogen receptors and breast cancer. Epidemiol. Rev. *8*, 42–59.

50. Liberzon, A., Birger, C., Thorvaldsdóttir, H., Ghandi, M., Tamayo, P., and Mesirov, J.P. (2015). The Molecular Signatures Database Hallmark Gene Set Collection. Cell Syst. *1*, 417–425. https://doi.org/10.1016/j.cels.2015.12.004.

51. Frasor, J., Danes, J.M., Komm, B., Chang, K.C.N., Lyttle, C.R., and Katzenellenbogen, B.S. (2003). Profiling of Estrogen Up- and Down-Regulated Gene Expression in Human Breast Cancer Cells: Insights into Gene Networks and Pathways Underlying Estrogenic Control of Proliferation and Cell Phenotype. Endocrinology *144*, 4562–4574. https://doi.org/10.1210/en.2003-0567.

52. Jagannathan, V., and Robinson-Rechavi, M. (2011). Meta-analysis of estrogen response in MCF-7 distinguishes early target genes involved in signaling and cell proliferation from later target genes involved in cell cycle and DNA repair. BMC Syst. Biol. *5*, 138–214.

53. Fritzsch, C., Baumgärtner, S., Kuban, M., Steinshorn, D., Reid, G., and Legewie, S. (2018). Estrogen-dependent control and cell-to-cell variability of transcriptional bursting. Mol. Syst. Biol. *14*, e7678. https://doi.org/10.15252/msb.20177678.

54. Rodriguez, J., Ren, G., Day, C.R., Zhao, K., Chow, C.C., and Larson, D.R. (2019). Intrinsic Dynamics of a Human Gene Reveal the Basis of Expression Heterogeneity. Cell *176*, 213–226.e18. https://doi.org/10.1016/j.cell.2018.11.026.

55. Shu, S., Lin, C.Y., He, H.H., Witwicki, R.M., Tabassum, D.P., Roberts, J.M., Janiszewska, M., Huh, S.J., Liang, Y., Ryan, J., et al. (2016). Response and resistance to BET bromodomain inhibitors in triple-negative breast cancer. Nature *529*, 413–417. https://doi.org/10.1038/nature16508.

56. Zhang, G., Zhao, Y., Liu, Y., Kao, L.-P., Wang, X., Skerry, B., and Li, Z. (2016). FOXA1 defines cancer cell specificity. Sci. Adv. *2*, e1501473. https://doi.org/10.1126/sciadv.1501473.

57. Kursa, M.B., and Rudnicki, W.R. (2010). Feature Selection with the Boruta Package. J. Stat. Software *36*, 1–13. https://doi.org/10.18637/jss.v036.i11.

58. Sigalova, O.M., Shaeiri, A., Forneris, M., Furlong, E.E., and Zaugg, J.B. (2020). Predictive features of gene expression variation reveal mechanistic link with differential expression. Mol. Syst. Biol. *16*, e9539.

59. Schier, A.C., and Taatjes, D.J. (2020). Structure and mechanism of the RNA polymerase II transcription machinery. Genes Dev. *34*, 465–488. https://doi.org/10.1101/gad.335679.119.

60. Wang, Z., Zang, C., Rosenfeld, J.A., Schones, D.E., Barski, A., Cuddapah, S., Cui, K., Roh, T.-Y., Peng, W., Zhang, M.Q., and Zhao, K. (2008). Combinatorial patterns of histone acetylations and methylations in the human genome. Nat. Genet. *40*, 897–903. https://doi.org/10.1038/ng.154.

61. Bauer, D.F. (1972). Constructing confidence sets using rank statistics. J. Am. Stat. Assoc. *67*, 687–690.

62. Gertz, J., Savic, D., Varley, K.E., Partridge, E.C., Safi, A., Jain, P., Cooper, G.M., Reddy, T.E., Crawford, G.E., and Myers, R.M. (2013). Distinct Properties of Cell-Type-Specific and Shared Transcription Factor Binding Sites. Mol. Cell *52*, 25–36. https://doi.org/10.1016/j.molcel.2013.08.037.

63. Iyer, V.R., Eisen, M.B., Ross, D.T., Schuler, G., Moore, T., Lee, J.C., Trent, J.M., Staudt, L.M., Hudson, J., Jr., Boguski, M.S., et al. (1999). The transcriptional program in the response of human fibroblasts to serum. Science *283*, 83–87. https://doi.org/10.1126/science.283.5398.83.

64. Gertz, J., Reddy, T.E., Varley, K.E., Garabedian, M.J., and Myers, R.M. (2012). Genistein and bisphenol A exposure cause estrogen receptor 1 to bind thousands of sites in a cell type-specific manner. Genome Res. *22*, 2153–2162. https://doi.org/10.1101/gr.135681.111.

65. Baudino, T.A., and Cleveland, J.L. (2001). The Max network gone mad. Mol. Cell Biol. *21*, 691–702. https://doi.org/10.1128/mcb.21.3.691-702.2001.

66. Agarwal, V., and Kelley, D.R. (2022). The genetic and biochemical determinants of mRNA degradation rates in mammals. Genome Biol. *23*, 245. https://doi.org/10.1186/s13059-022-02811-x.

67. Carleton, J.B., Berrett, K.C., and Gertz, J. (2017). Multiplex Enhancer Interference Reveals Collaborative Control of Gene Regulation by Estrogen Receptor α-Bound Enhancers. Cell Syst *5*, 333–344.e335. https://doi.org/10.1016/j.cels.2017.08.011.

68. Urrutia, R. (2003). KRAB-containing zinc-finger repressor proteins. Genome Biol. *4*, 231. https://doi.org/10.1186/gb-2003-4-10-231.

69. Ginley-Hidinger, M., Carleton, J.B., Rodriguez, A.C., Berrett, K.C., and Gertz, J. (2019). Sufficiency analysis of estrogen responsive enhancers using synthetic activators. Life Sci. Alliance *2*, e201900497. https://doi.org/10.26508/lsa.201900497.

70. Bignotti, E., Zanotti, L., Calza, S., Falchetti, M., Lonardi, S., Ravaggi, A., Romani, C., Todeschini, P., Bandiera, E., Tassi, R.A., et al. (2012). Trop-2 protein overexpression is an independent marker for predicting disease recurrence in endometrioid endometrial carcinoma. BMC Clin. Pathol. *12*, 22. https://doi.org/10.1186/1472-6890-12-22.

71. Shvartsur, A., and Bonavida, B. (2015). Trop2 and its overexpression in cancers: regulation and clinical/therapeutic implications. Genes & Cancer *6*, 84–105. https://doi.org/10.18632/genesandcancer.40.

72. Brennecke, P., Anders, S., Kim, J.K., Kołodziejczyk, A.A., Zhang, X., Proserpio, V., Baying, B., Benes, V., Teichmann, S.A., Marioni, J.C., and Heisler, M.G. (2013). Accounting for technical noise in single-cell

CellPress
OPEN ACCESS

RNA-seq experiments. Nat. Methods *10*, 1093–1095. https://doi.org/10.1038/nmeth.2645.

73. Benayoun, B.A., Pollina, E.A., Ucar, D., Mahmoudi, S., Karra, K., Wong, E.D., Devarajan, K., Daugherty, A.C., Kundaje, A.B., Mancini, E., et al. (2014). H3K4me3 breadth is linked to cell identity and transcriptional consistency. Cell *158*, 673–688. https://doi.org/10.1016/j.cell.2014.06.027.
74. Fraser, L.C., Dikdan, R.J., Dey, S., Singh, A., and Tyagi, S. (2021). Reduction in Gene Expression Noise by Targeted Increase in Accessibility at Gene Loci. Proceedings of the National Academy of Sciences *118*, e2018640118.
75. Ham, L., Jackson, M., and Stumpf, M.P. (2021). Pathway dynamics can delineate the sources of transcriptional noise in gene expression. Elife *10*, e69324. https://doi.org/10.7554/eLife.69324.
76. Boe, R.H., Ayyappan, V., Schuh, L., and Raj, A. (2022). Allelic correlation is a marker of trade-offs between barriers to transmission of expression variability and signal responsiveness in genetic networks. Cell Syst. *13*, 1016–1032.e6.
77. Borriello, E., Walker, S.I., and Laubichler, M.D. (2020). Cell phenotypes as macrostates of the GRN dynamics. J. Exp. Zool. B Mol. Dev. Evol. *334*, 213–224. https://doi.org/10.1002/jez.b.22938.
78. Zhang, J., Zhu, W., Wang, Q., Gu, J., Huang, L.F., and Sun, X. (2019). Differential regulatory network-based quantification and prioritization of key genes underlying cancer drug resistance based on time-course RNA-seq data. PLoS Comput. Biol. *15*, e1007435.
79. Tani, H., Mizutani, R., Salam, K.A., Tano, K., Ijiri, K., Wakamatsu, A., Isogai, T., Suzuki, Y., and Akimitsu, N. (2012). Genome-wide determination of RNA stability reveals hundreds of short-lived noncoding transcripts in mammals. Genome Res. *22*, 947–956.
80. Reddy, T.E., Pauli, F., Sprouse, R.O., Neff, N.F., Newberry, K.M., Garabedian, M.J., and Myers, R.M. (2009). Genomic determination of the glucocorticoid response reveals unexpected mechanisms of gene regulation. Genome Res. *19*, 2163–2171.
81. Hassig, C.A., Fleischer, T.C., Billin, A.N., Schreiber, S.L., and Ayer, D.E. (1997). Histone deacetylase activity is required for full transcriptional repression by mSin3A. Cell *89*, 341–347. https://doi.org/10.1016/s0092-8674(00)80214-7.
82. Langmead, B., Trapnell, C., Pop, M., and Salzberg, S.L. (2009). Ultrafast and memory-efficient alignment of short DNA sequences to the human genome. Genome Biol. *10*, R25.
83. Li, H., Handsaker, B., Wysoker, A., Fennell, T., Ruan, J., Homer, N., Marth, G., Abecasis, G., and Durbin, R.; 1000 Genome Project Data Processing Subgroup (2009). The Sequence Alignment/Map format and SAMtools. Bioinformatics *25*, 2078–2079. https://doi.org/10.1093/bioinformatics/btp352.
84. Zhang, Y., Liu, T., Meyer, C.A., Eeckhoute, J., Johnson, D.S., Bernstein, B.E., Nusbaum, C., Myers, R.M., Brown, M., Li, W., and Liu, X.S. (2008). Model-based analysis of ChIP-Seq (MACS). Genome Biol. *9*, R137–R139.
85. Mumbach, M.R., Rubin, A.J., Flynn, R.A., Dai, C., Khavari, P.A., Greenleaf, W.J., and Chang, H.Y. (2016). HiChIP: efficient and sensitive analysis of protein-directed genome architecture. Nat. Methods *13*, 919–922.
86. Servant, N., Varoquaux, N., Lajoie, B.R., Viara, E., Chen, C.-J., Vert, J.-P., Heard, E., Dekker, J., and Barillot, E. (2015). HiC-Pro: an optimized and flexible pipeline for Hi-C data processing. Genome Biol. *16*, 259–311.
87. Lareau, C.A., and Aryee, M.J. (2018). hichipper: a preprocessing pipeline for calling DNA loops from HiChIP data. Nat. Methods *15*, 155–156.
88. Mahat, D.B., Kwak, H., Booth, G.T., Jonkers, I.H., Danko, C.G., Patel, R.K., Waters, C.T., Munson, K., Core, L.J., and Lis, J.T. (2016). Base-pair-resolution genome-wide mapping of active RNA polymerases using precision nuclear run-on (PRO-seq). Nat. Protoc. *11*, 1455–1476. https://doi.org/10.1038/nprot.2016.086.
89. Martin, M. (2011). Cutadapt Removes Adapter Sequences from High-Throughput Sequencing Reads*17*, p. 3. https://doi.org/10.14806/ej.17.1.200.
90. Hannon, G.J. (2010). FASTX-Toolkit FASTQ/A short-reads pre-processing tools. http://hannonlab.cshl.edu/fastx_toolkit/.
91. Langmead, B., and Salzberg, S.L. (2012). Fast gapped-read alignment with Bowtie 2. Nat. Methods *9*, 357–359.
92. Mcginnis, C.S., Patterson, D.M., Winkler, J., Conrad, D.N., Hein, M.Y., Srivastava, V., Hu, J.L., Murrow, L.M., Weissman, J.S., Werb, Z., et al. (2019). MULTI-seq: sample multiplexing for single-cell RNA sequencing using lipid-tagged indices. Nat. Methods *16*, 619–626. https://doi.org/10.1038/s41592-019-0433-8.
93. Single Cell Suspensions from Cultured Cell Lines for Single Cell RNA Sequencing (2017). 10x Genomics *Document Number CG00054 Rev B*.
94. Stuart, T., Butler, A., Hoffman, P., Hafemeister, C., Papalexi, E., Mauck, W.M., Hao, Y., Stoeckius, M., Smibert, P., and Satija, R. (2019). Comprehensive Integration of Single-Cell Data. Cell *177*, 1888–1902.e21. https://doi.org/10.1016/j.cell.2019.05.031.
95. R Core Team (2013). R: A Language and Environment for Statistical Computing. MSOR Connections, 1.
96. Xie, Z., Bailey, A., Kuleshov, M.V., Clarke, D.J.B., Evangelista, J.E., Jenkins, S.L., Lachmann, A., Wojciechowicz, M.L., Kropiwnicki, E., Jagodnik, K.M., et al. (2021). Gene set knowledge discovery with Enrichr. Curr. Protoc. *1*, e90.
97. Wood, S.N. (2006). Generalized Additive Models: An Introduction with R (Chapman and Hall/CRC).
98. Satija, R., Farrell, J.A., Gennert, D., Schier, A.F., and Regev, A. (2015). Spatial reconstruction of single-cell gene expression data. Nat. Biotechnol. *33*, 495–502.
99. Pruitt, K.D., Brown, G.R., Hiatt, S.M., Thibaud-Nissen, F., Astashyn, A., Ermolaeva, O., Farrell, C.M., Hart, J., Landrum, M.J., McGarvey, K.M., et al. (2014). RefSeq: an update on mammalian reference sequences. Nucleic Acids Res. *42*, D756–D763. https://doi.org/10.1093/nar/gkt1114.
100. Kursa, M.B., and Rudnicki, W.R. (2010). Feature selection with the Boruta package. J. Stat. Software *36*, 1–13.
101. Breiman, L., Friedman, J.H., Olshen, R.A., and Stone, C.J. (2017). Classification and Regression Trees (Routledge).
102. Cheng, A.W., Wang, H., Yang, H., Shi, L., Katz, Y., Theunissen, T.W., Rangarajan, S., Shivalila, C.S., Dadon, D.B., and Jaenisch, R. (2013). Multiplexed activation of endogenous genes by CRISPR-on, an RNA-guided transcriptional activator system. Cell Res. *23*, 1163–1171.
103. Benchling (2020). https://www.benchling.com/.
104. Cleveland, W.S., Grosse, E., and Shyu, W.M. (2017). Local regression models. In Statistical models in S (Routledge), pp. 309–376.
105. Kim, D., Paggi, J.M., Park, C., Bennett, C., and Salzberg, S.L. (2019). Graph-based genome alignment and genotyping with HISAT2 and HISAT-genotype. Nat. Biotechnol. *37*, 907–915. https://doi.org/10.1038/s41587-019-0201-4.
106. Kent, W.J., Sugnet, C.W., Furey, T.S., Roskin, K.M., Pringle, T.H., Zahler, A.M., and Haussler, D. (2002). The human genome browser at UCSC. Genome Res. *12*, 996–1006.
107. Liao, Y., Smyth, G.K., and Shi, W. (2014). featureCounts: an efficient general purpose program for assigning sequence reads to genomic features. Bioinformatics *30*, 923–930.
108. Love, M., Anders, S., and Huber, W. (2014). Differential analysis of count data–the DESeq2 package. Genome Biol. *15*, 10–1186.

STAR★METHODS

KEY RESOURCES TABLE

REAGENT or RESOURCE	SOURCE	IDENTIFIER
Antibodies		
anti-H3K27ac	Abcam	Cat# ab4729; RRID: AB_2118291
anti-MAX	Santa Cruz Biotechnology	Cat# sc-8011; RRID: AB_627913
anti-LSD1	Abcam	Cat# ab17721; RRID: AB_443964
anti-TAF1	Santa Cruz Biotechnology	Cat# sc-735; RRID: AB_671202
anti-c-MYC	Santa Cruz Biotechnology	Cat# sc-40; RRID: AB_627268
anti-H3K4me3	Cell Signaling Technologies	Cat# 9751S; RRID: AB_2616028
anti-H3K4me1	Cell Signaling Technologies	Cat# 5326S; RRID: AB_10695148
anti-SIN3A	Hassig et al.[81]	N/A
anti-RARA	Santa Cruz Biotechnology	Cat# sc-515796; RRID: AB_2910553
anti-c-JUN	BD Biosciences	Cat# 558036; RRID: AB_2249448
Deposited data		
ChIP-seq	This study	GEO: GSE227241
Hi-ChIP	This study	GEO: GSE227242
PRO-seq	This study	GEO: GSE227243
scRNA-seq	This study	GEO: GSE227244
RNA-seq	This study	GEO: GSE245120
Additional publicly available data	See Table S3	N/A
Experimental models: Cell lines		
Ishikawa	Sigma-Aldrich	Cat# 99040201-1VL
T-47D	ATCC	Cat# HTB-133
LNCaP	Cancer Cell Line Encyclopedia	N/A
Oligonucleotides		
Oligo lists	This paper; see Tables S1 and S2	N/A
Software and algorithms		
HiC-Pro	Servant et al.[86]	https://github.com/nservant/HiC-Pro
Hichipper	Lareau and Aryee[87]	https://github.com/aryeelab/hichipper
FASTX	Hannon[90]	http://hannonlab.cshl.edu/fastx_toolkit/index.html
cutadapt	Martin[89]	https://github.com/marcelm/cutadapt
demultiplex R package	McGinnis et al.[92]	https://rdrr.io/github/chris-mcginnis-ucsf/MULTI-seq/
Seurat v3	Stuart et al.[94]	https://cran.r-project.org/src/contrib/Archive/Seurat/
Boruta	Kursa and Rudnicki[100]	https://cran.r-project.org/web/packages/Boruta/index.html
HISAT2	Kim et al.[105]	https://daehwankimlab.github.io/hisat2/
SAMtools	Li et al.[83]	https://github.com/samtools/samtools
DESeq2	Love et al.[108]	https://github.com/thelovelab/DESeq2
featureCounts	Liao et al.[107]	https://bioconductor.org/packages/release/bioc/html/Rsubread.html

RESOURCES AVAILABILITY

Lead contact

Further information and requests for resources and reagents should be directed to and will be fulfilled by the lead contact, Jason Gertz (jay.gertz@hci.utah.edu).

Materials availability

This study did not generate any new unique reagents.

CellPress
OPEN ACCESS

Data and code availability

ChIP-seq, HiChIP, PRO-seq, RNA-seq, and scRNA-seq data are available at the Gene Expression Omnibus under accession GSE227245. Any additional information required to reanalyze the data reported in this paper is available from the lead contact upon request.

EXPERIMENTAL MODEL AND SUBJECT DETAILS

Cell lines

T-47D cells were obtained from ATCC and Ishikawa cells were obtained from Sigma-Aldrich. Both cell lines were cultured in RPMI 1640 medium (Gibco) with 10% fetal bovine serum (Gibco) and 1% penicillin–streptomycin (Gibco). LNCaP cells were obtained from the Cancer Cell Line Encyclopedia (CCLE) and cultured in RPMI media with 10% FBS supplemented. Cells were incubated at 37°C with 5% CO_2. 5 days before estrogen inductions, cells were transferred to hormone-depleted media consisting of phenol red-free RPMI (Gibco) with 10% charcoal-dextran-stripped fetal bovine serum (Sigma-Aldrich) and 1% penicillin–streptomycin (Gibco). Cell authentication was not performed as part of this study.

METHOD DETAILS

ChIP-seq

After 5 days in hormone-depleted media, cells were plated in 15cm dishes at approximately 60% confluency 1 day before estrogen induction. Cells were treated with vehicle (DMSO) or E2 at a final concentration of 10nM for either 1 h for transcription factor ChIP-seqs, or 8 h for histone marker ChIP-seqs. ChIP and library preparation was performed as previously described.[80] Antibodies used for this study were MAX (Santa Cruz, sc-8011), LSD1 (Abcam, ab17721), TAF1 (Santa Cruz, sc-735), c-MYC (Santa Cruz, sc-40), H3K4me3 (Cell Signaling, 9751S), H3K4me1 (Cell Signaling, 5326S), SIN3A (produced as previously described),[81] RARA (Santa Cruz, sc-515796) and c-JUN (BD Biosciences, 558036). Libraries were sequenced using either an Illumina HiSeq 2500 or Illumina NovaSeq 6000 as single- or paired-end 50 bp reads, then aligned to hg19 using bowtie with parameters -m 1 –t –best -q -S -l 32 -e 80 -n 2.[82] Signal intensity was extracted from bam files using samtools view with parameter -c.[83] In the cases where peaks were called, peak calling was done using Macs2 with the default q-value cutoff of 0.05 and mfold ratio between 15 and 100.[84]

H3K27ac HiChIP

HiChIP experiments were performed as previously described[85] using an antibody that recognizes H3K27ac (Abcam, ab4729). Ishikawa cells were treated with either 10 nM E2 for 1 h or DMSO as a vehicle control. HiChIP in Ishikawa cells was conducted using restriction enzyme DpnII (New England Biolabs). Crosslinked chromatin was sonicated using an EpiShear probe-in sonicator (Active Motif) with three cycles of 30 s at an amplitude of 40% with 30 s rest between cycles. HiChIP libraries were sequenced on NovaSeq 6000 as paired end 50 base pair reads to an average depth of 300–400 million read-pairs per sample.

Experiments in T-47D and LNCaP cells were conducted using restriction enzyme MboI (New England Biolabs). Crosslinked chromatin was sonicated using Covaris E220 with the settings of fill level = 10, duty cycle = 5, PIP = 140, cycles per burst = 200, time = 4 min. HiChIP libraries were sequenced on HiSeq 2500 as paired end 75 base pair reads to ~50 million read pairs per sample.

Reads were aligned to human hg19 reference genome using HiC-Pro.[86] Hichipper[87] was used to perform restriction site bias-aware modeling of the output from HiC-Pro and to call interaction loops. In Ishikawa cells, DMSO and E2 treated HiChIP loops were combined to identify all possible putative enhancers. In all datasets, loops with less than 3 reads or FDR $\geq$ 0.05 were filtered out.

PRO-seq

PRO-seq libraries were generated as described in Mahat et al., 2016.[88] Briefly, Ishikawa and T-47D cells were grown in hormone-depleted RPMI for five days, then $2x10^6$ cells were plated into two 10 cm dishes per condition with RPMI lacking phenol red supplemented with 10% charcoal/dextran-stripped FBS and penicillin. Cells were treated with vehicle (DMSO) or 10 nM E2 for 45 min, then permeabilized for 5 min with permeabilization buffer [10 mM Tris-HCl, pH 7.4; 300 mM sucrose; 10 mM KCl; 5 mM MgCl2; 1 mM EGTA; 0.05% Tween 20; 0.1% NP40 substitute; 0.5 mM DTT, protease inhibitor cocktail ml(Roche); and SUPERaseIn RNase Inhibitor (Ambion)]. The nuclear run-on was performed by adding permeabilized cells to run-on mixture [final composition was 5 mM Tris, pH 8.0; 25 mM MgCl2; 0.5 mM DTT; 150 mM KCl; 200 μM rATP; 200 μM rGTP; 20 μM biotin-11-rCTP (Perkins Elmer); 20 μM biotin-11-rUTP (Perkins Elmer); 1 U/μL SUPERase In RNase Inhibitor (Ambion); 0.5% Sarkosyl], then incubating at 37°C for 5 min. RNA was extracted with Trizol LS (Ambion), fragmented with 0.2 N NaOH for 8 min on ice, then neutralized with 0.5 M Tris, pH 6.8, followed by buffer exchange with a P-30 column (Bio-Rad). Biotinylated RNAs were enriched with Dynabeads M280 Streptavidin (Invitrogen), then RNA was extracted with Trizol (Ambion), followed by 3′ adapter ligation using T4 RNA ligase (NEB). Biotinylated RNAs were enriched for a second time, followed by 5′ cap repair with RppH (NEB) and 5′ hydroxyl repair with PNK (NEB). The 5′ adapter was ligated with T4 RNA ligase (NEB), followed by a third biotinylated RNA enrichment. Reverse transcription was performed with the RP1 primer. Samples were PCR amplified for 13 cycles, then cleaned up with Agencourt AMPure XP beads (Beckman Coulter). Libraries were sequenced on an Illumina HiSeq 2500, generating a 50nt read. Reads were processed using cutadapt[89] with parameters -a

TGGAATTCTCGGGTGCCAAGG –cut 7 –length 42 -m 21. Reverse complement sequences were generated using fastx_reverse_complement from the FASTX toolkit (v 0.0.13).[90] Reads were then aligned to hg19 with bowtie2[91] in end-to-end mode, and non-uniquely aligned reads were discarded.

scRNA-seq

Cells were treated with 10nM E2 for 0 (vehicle treated), 2, 4, and 8 h. To mitigate technical batch effects, cells were labeled via MULTI-seq as previously described.[92] Cells from different time points were mixed and then prepared according to the 10x Genomics sample prep user guide.[93] Cells were separated into single cell emulsions using the 10x Genomics Chromium Controller with a targeted recovery of 10,000 cells. Sequencing libraries were prepared using the 10X Genomics Next GEM Single Cell 3′ Gene Expression Library prep v3.1. Sequencing was performed on an Illumina NovaSeq 6000 with 150bp read length. Sequencing output was processed from reads to counts using the 10x Genomics Cell Ranger v3.1.0 pipeline. MULTI-seq calls were processed using the demultiplex R package[92] and mapped back to the E2 time points. Counts were log normalized using the Seurat v3 R package,[94] then filtered using custom cutoffs. For Ishikawa cells, cell filtering criteria were unique reads between 8500 and 35,000, unique genes between 2700 and 6000, and percent mitochondrial reads less than 7%. For T-47D cells, cell filtering criteria were unique reads between 7500 and 40,000, unique genes between 1500 and 6000, and percent mitochondrial reads less than 20%. Genes are filtered to have a mean greater than 0.01 across all time points.

scRNA-seq analysis: Classification of trajectory and noise levels

Computational analysis of trajectory and noise levels were conducted using R.[95] Trajectory classification was done using a two-sided Wilcoxon test[61] to find genes whose single cell distributions significantly change at different time points compared to the 0h time point. Genes that change significantly by 2 h are classified as either "Early Up" or "Early Down". Genes with changes seen at 4 or 8 h are classified as "Late". It is important to note that genes were called differential without a fold change cutoff. The statistical power of scRNA-seq allowed for the identification of differentially expressed genes with smaller fold changes, but appreciable absolute changes; however, due to technical limitations of scRNA-seq our results may be affected by technical variation or drop out of lower expressed genes. To select control genes with similar mean distributions to those genes that are regulated by E2, we used a stratified sampling approach to select control genes that are not significantly regulated. Genes classified into Early and Late Up categories were analyzed by EnrichR[96] using the MSigDB Hallmark 2020 gene annotations.

Our noise metric is defined as the residuals from a generalized additive model (GAM) regression fitted to the CV vs. mean for all genes. Regression was performed on log2(CV + 1) vs. mean curve using the gam function from the mgcv R package with formula y $\sim$ s(x, bs = "cs").[97] Residuals were then transformed back to the original scale. Noise levels were determined using the GAM-adjusted CV. We chose the GAM method as it removed most of the mean-noise relationship (Figure S6G), as desired, compared to SCTransform standardized variance and residual variance as shown in Figures S5C and S5D. Overall, we found that genes were binned as High noise more consistently across the three methods (average fraction overlap of 0.82) than Medium (0.67) or Low noise (0.58); however, less than 1% of genes were classified as High noise by one method and Low noise by another. For this comparison of noise metrics, we used the HVFInfo function with selection.method as "vst" or "sctransform" in Seurat.[98] To account for the different ranges in noise at different mean levels, genes were binned by mean and then into 3 groups of noise levels by quantile. The top 20% and bottom 20% of genes in each quantile were labeled as "High" and "Low" noise, respectively.

Feature importance analysis

Promoters were defined as 500bp regions centered on the transcriptional start site, as annotated in the RefSeq database.[99] Enhancers were called using H3K27ac HiChIP data and H3K27ac ChIP-seq peaks. Enhancers were defined as H3K27ac peaks within HiChIP anchors that loop to the promoter. Integrated signal for each promoter and enhancer was collected from all datasets using samtools view -c.[83] Z-scores were calculated across all genes for input to feature ranking algorithms. An enhancer score was calculated to account for signal at multiple enhancers, using the formula

$$\sum_{1}^{n} log_2(s+1)$$

where n represents the number of enhancers associated with a gene and s represents the *Z* score of integrated genomic signal at each enhancer.

Number of enhancers was defined as the number of H3K27ac peaks that loop to the promoter, as determined by HiChIP. Number of ERBS was calculated as the number of enhancers that overlapped with ER ChIP-seq peaks. Feature ranking was performed using the Boruta package in R[100] with default parameters and 100 maximum iterations. Gene length was calculated from RefSeq transcript annotations.[99] An example decision tree was determined using the rpart function with parameters minbucket = 50 and cp = 0.007.[101] Average enhancer score and promoter signal was calculated using "confirmed" variables from Boruta analysis. The average of *Z* score signal for confirmed variables was taken for all variables associated with either the promoter or enhancer, not including number of enhancers, number of ERBS, or gene length.

Generation of stable dCas9-VP16(10x) cell lines

Ishikawa cells were plated in 6-well plates at 60% confluency. Cells were transfected with Addgene plasmid 48227 (a gift from Rudolf Jaenisch)[102] containing dCas9-VP16(10x) with a P2A linker and neomycin resistance gene. Fugene HD (Promega) was used for transfection at a 3:1 reagent:DNA ratio. dCas9-VP16(10x) plasmid was linearized with restriction enzyme AflII (New England Biolabs R0520S). Successful integration of the dCas9-VP16(10x) plasmid was selected for using G418 (Thermo Scientific) at a concentration of 800 μg/mL for approximately 2 weeks. Successful expression of the dCas9 plasmid was verified using qPCR for dCas9 as well as qPCR for successful activation of a control gene, *IL1RN*. Cells were then maintained at a lower concentration of 400 μg/mL G418.

gRNA design and transfection

gRNAs were designed using the Benchling gRNA design tool.[103] 4 gRNAs were designed per targeted region. gRNAs were cloned into plasmids as previously described.[67] gRNA sequence and adjacent PAM are listed in Table S1. Prior to transfection, Ishikawa cells were plated in 48-well plates at 80,000 cells/well. 24 h after plating, gRNAs were transfected into cell using Fugene HD (Promega) at a manufacturer suggested 3:1 reagent:DNA ratio. gRNA transfection was selected for using 1 μg/mL puromycin. 8-h E2 time courses were started roughly 24 h after addition of puromycin.

RNA isolation and qPCR gene expression analysis

Cells were lysed with Buffer RLT Plus (Qiagen) containing 1% beta-mercaptoethanol (Sigma). RNA was purified using the ZR-96-well Quick-RNA kit (Zymo Research). Gene expression was measured using qPCR with reagents from the Power SYBR Green RNA-to-Ct 1-step kit (Applied Biosystems), 50ng RNA per reaction, and 40 cycles on a CFX Connect light cycler (BioRad). qPCR primers are listed in Table S2. Relative expression was calculated using the ΔΔCt method with *CTCF* as a reference gene and cells where the same dCas9 fusion is targeted to the *IL1RN* promoter as the controls. Best fit lines were determined using the loess function in R[104] and formula $y \sim x$. Half maximal values were calculated using the maximum at any time point. The first time point at which the loess regression reaches a half maximal value is recorded as the time to half maximal. Differentials of the loess regression were also calculated using R to examine how the slope of each trajectory changes over time. Slope analysis was binned into "targeted" and "control" groups to analyze the general effect of dCas9 manipulation at regulatory regions of the respective gene.

Bulk RNA-seq experiments and analysis

Cells were treated with 10nM E2 for 0 (vehicle treated), 2, 4, and 8 h. Following treatments, cells were lysed with buffer RLT Plus (Qiagen) containing 1% beta-mercaptoethanol (Sigma-Aldrich). RNA was extracted and purified using a Quick RNA Mini Prep kit (Zymo Research). NEBNext Ultra II Directional RNA Library Prep kit with poly(A) mRNA isolation was used to construct RNA-seq libraries according to the manufacturer's instructions (NEB). Sequencing reads were aligned to hg19 build of the human genome using HISAT2.[105] SAMtools[83] was used to convert SAM files to BAM files. Genes were defined by the University of California Santa Cruz (UCSC) Known Genes[106] and reads that mapped to known genes were assigned with featureCounts.[107] Read counts were normalized and analyzed for differential expression via DESeq2.[108] Genes that were differentially expressed between the 0-h and 2-h time points were defined as Early, while all other differentially expressed genes were defined as Late.

Co-expression analysis

Spearman correlation values were calculated using the cor function in R. Pairs were defined either by looping data, or for a set of genes, as all possible combinations of genes in a list. Control pairings for genes that were looped together were generated by randomly shuffling the pairings. Control genes in the time course correlation analysis refer to the pairs within the set of timing control genes (defined above) where stratified sampling was used to replicate the same mean distribution as regulated genes.

QUANTIFICATION AND STATISTICAL ANALYSIS

All statistical details are indicated in the STAR Methods, Results, or figure legends.

Cell Genomics, Volume *4*

Supplemental information

Cis-regulatory control of transcriptional timing and noise in response to estrogen

Matthew Ginley-Hidinger, Hosiana Abewe, Kyle Osborne, Alexandra Richey, Noel Kitchen, Katelyn L. Mortenson, Erin M. Wissink, John Lis, Xiaoyang Zhang, and Jason Gertz

Supplementary Information

Cis-regulatory control of transcriptional timing and noise in response to estrogen

Matthew Ginley-Hidinger[1,2], Hosiana Abewe[1,3], Kyle Osborne[1,3], Alexandra Richey[1,2], Noel Kitchen[1,3], Katelyn L. Mortenson[1,3], Erin M. Wissink[4], John Lis[4], Xiaoyang Zhang[1,3], Jason Gertz[1,2,3,*]

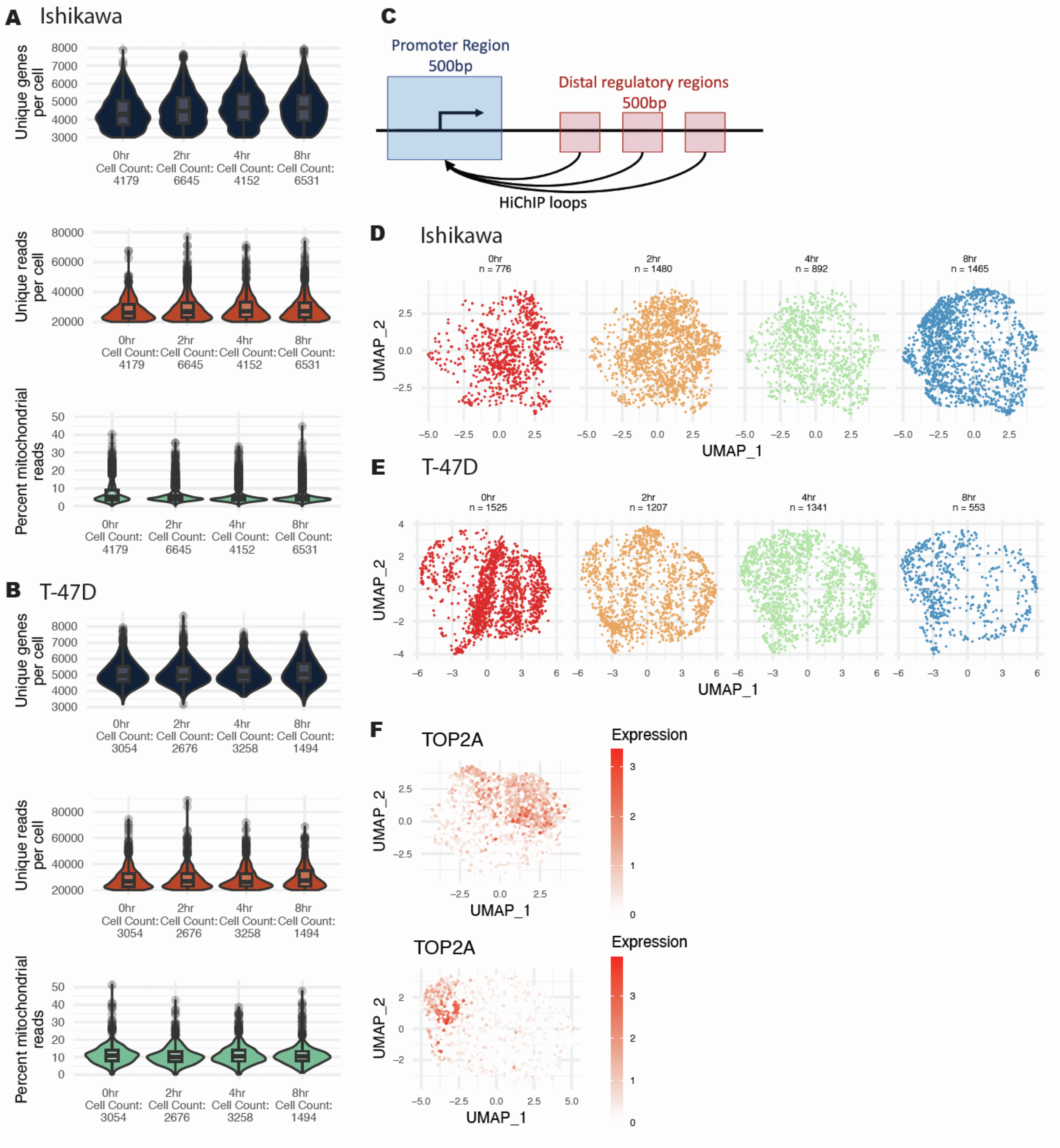

Supplementary Figure 1 (related to Figures 1 and 2). QC plots for scRNA-seq and diagram of *cis*-regulatory model. (A-B) Distribution of reads, genes, and percent mitochondrial reads per cell, separated by timepoint, are shown for (A) Ishikawa and (B) T-47D. Data shown is prior to filtering. (C) A model shows how signal intensity was collected for promoters and enhancers using HiChIP loops to identify enhancers. (D-E) UMAP dimensionality reduction plots for (D) Ishikawa and (E) T-47D show temporal progression of cells treated with E2. Each point represents a cell and timepoints are post 10 nM E2 induction. Cell numbers represent post-filtering. All cells for a cell line were used to create the original UMAP projections and the space was held constant when plotting each time point. (F) UMAP representation of cell-cycle marker gene *TOP2A,* which peaks in the G2/M phase, is shown in Ishikawa (top) and T-47D (bottom) to highlight the location of proliferating cells.

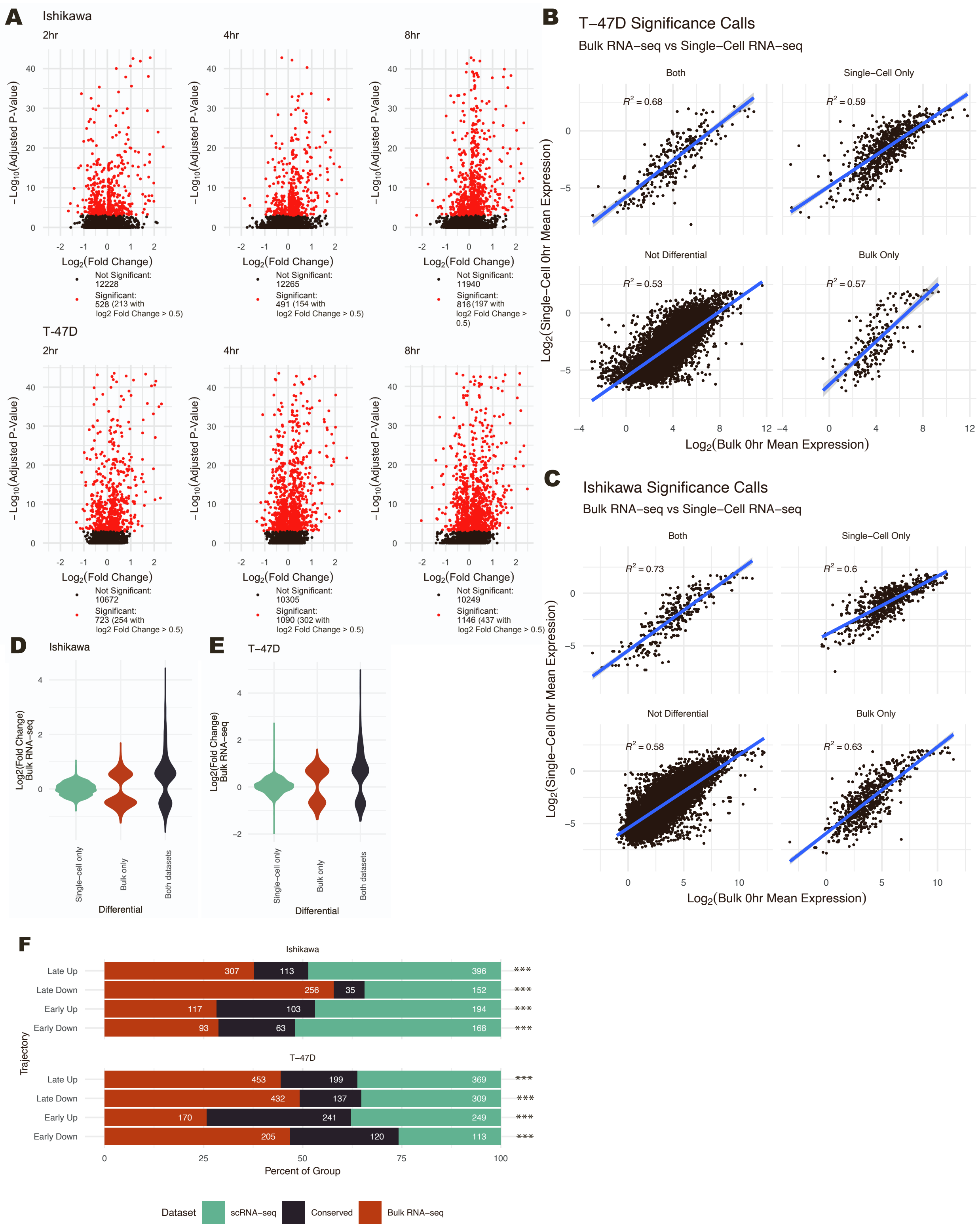

A
Ishikawa
2hr
4hr
8hr
−Log10(Adjusted P-Value)
Log2(Fold Change)
Not Significant: 12228
Significant: 528 (213 with log2 Fold Change > 0.5)
Not Significant: 12265
Significant: 491 (154 with log2 Fold Change > 0.5)
Not Significant: 11940
Significant: 816 (197 with log2 Fold Change > 0.5)
T-47D
Not Significant: 10672
Significant: 723 (254 with log2 Fold Change > 0.5)
Not Significant: 10305
Significant: 1090 (302 with log2 Fold Change > 0.5)
Not Significant: 10249
Significant: 1146 (437 with log2 Fold Change > 0.5)
B
T−47D Significance Calls
Bulk RNA−seq vs Single−Cell RNA−seq
Both
Single−Cell Only
Not Differential
Bulk Only
R² = 0.68
R² = 0.59
R² = 0.53
R² = 0.57
Log2(Single−Cell 0hr Mean Expression)
Log2(Bulk 0hr Mean Expression)
C
Ishikawa Significance Calls
Bulk RNA−seq vs Single−Cell RNA−seq
Both
Single−Cell Only
Not Differential
Bulk Only
R² = 0.73
R² = 0.6
R² = 0.58
R² = 0.63
Log2(Single−Cell 0hr Mean Expression)
Log2(Bulk 0hr Mean Expression)
D
Ishikawa
E
T−47D
Log2(Fold Change) Bulk RNA−seq
Single−cell only
Bulk only
Both datasets
Differential
F
Ishikawa
Late Up 307 113 396 ***
Late Down 256 35 152 ***
Early Up 117 103 194 ***
Early Down 93 63 168 ***
T−47D
Late Up 453 199 369 ***
Late Down 432 137 309 ***
Early Up 170 241 249 ***
Early Down 205 120 113 ***
Trajectory
Percent of Group
Dataset
scRNA−seq
Conserved
Bulk RNA−seq

Supplementary Figure 2 (related to Figure 2). Analysis of differential gene expression over time. (A) Differential gene expression -$\log_{10}$(Wilcoxon p-values) are plotted against $\log_2$(fold change) for all genes at each timepoint vs 0 hours. Significantly differential genes are highlighted in red. Data is shown for Ishikawa (top) and T-47D (bottom) cells. (B-C) Average Reads Per Million from scRNA-seq are correlated to bulk RNA-seq RPM in both T-47D (B) and Ishikawa (C) cells, shown on a log scale. Genes are separated into groups depending on whether they are called significant in bulk RNA-seq, scRNA-seq, or both. (D-E) Log_2 fold change distribution is shown for differential genes called by scRNA-seq analysis or bulk RNA-seq analysis in Ishikawa (D) and T-47D (E) cells. (F) Trajectory analysis in bulk RNA-seq vs scRNA-seq reveals a significant overlap of genes classified into each trajectory. P-values are calculated using hypergeometric tests (***: $p < 1\text{x}10^{-28}$).

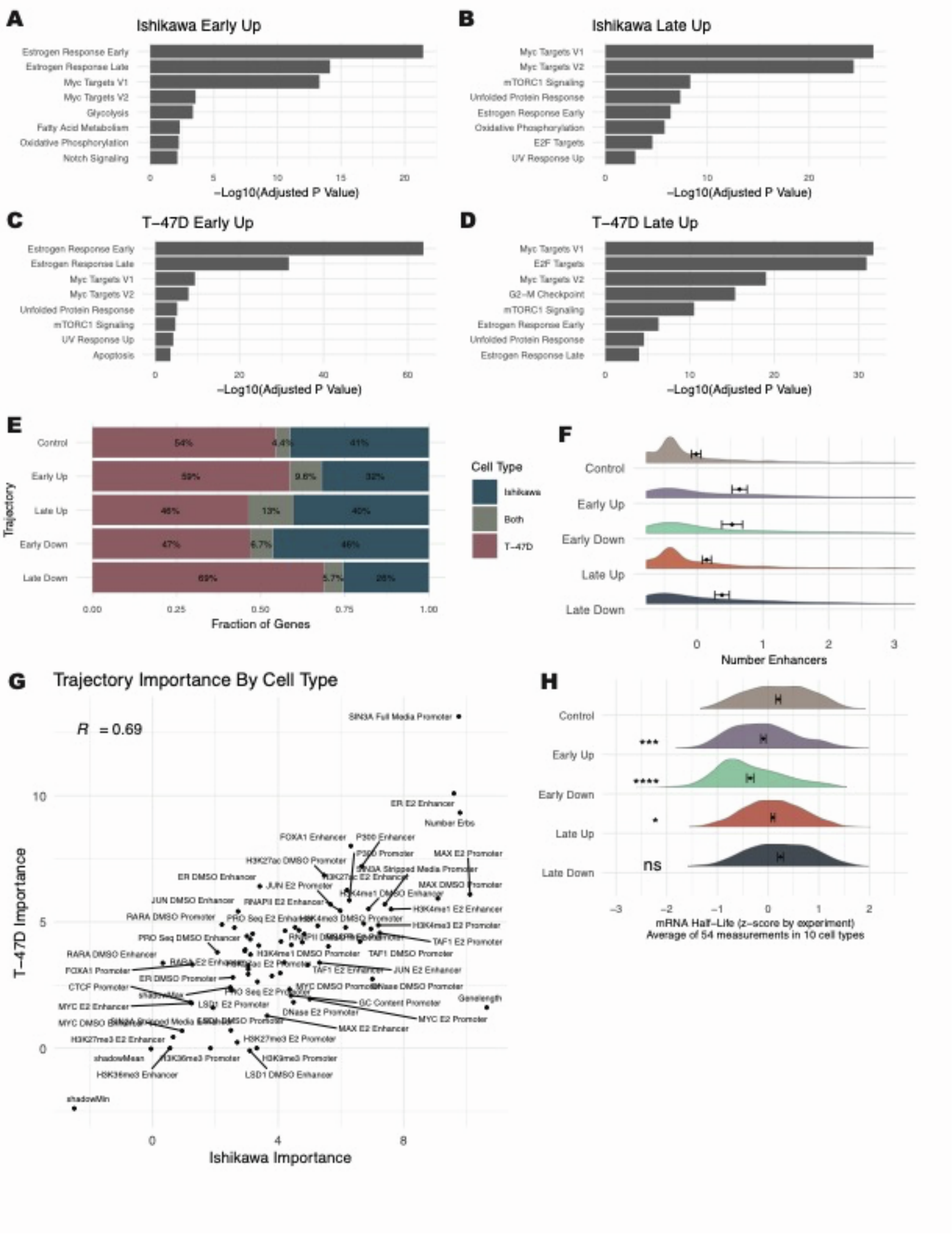

A
Ishikawa Early Up
Estrogen Response Early
Estrogen Response Late
Myc Targets V1
Myc Targets V2
Glycolysis
Fatty Acid Metabolism
Oxidative Phosphorylation
Notch Signaling
0 5 10 15 20
−Log10(Adjusted P Value)
B
Ishikawa Late Up
Myc Targets V1
Myc Targets V2
mTORC1 Signaling
Unfolded Protein Response
Estrogen Response Early
Oxidative Phosphorylation
E2F Targets
UV Response Up
0 10 20
−Log10(Adjusted P Value)
C
T−47D Early Up
Estrogen Response Early
Estrogen Response Late
Myc Targets V1
Myc Targets V2
Unfolded Protein Response
mTORC1 Signaling
UV Response Up
Apoptosis
0 20 40 60
−Log10(Adjusted P Value)
D
T−47D Late Up
Myc Targets V1
E2F Targets
Myc Targets V2
G2−M Checkpoint
mTORC1 Signaling
Estrogen Response Early
Unfolded Protein Response
Estrogen Response Late
0 10 20 30
−Log10(Adjusted P Value)
E
Trajectory
Control
Early Up
Late Up
Early Down
Late Down
0.00 0.25 0.50 0.75 1.00
Fraction of Genes
Cell Type
Ishikawa
Both
T−47D
F
Control
Early Up
Early Down
Late Up
Late Down
0 1 2 3
Number Enhancers
G
Trajectory Importance By Cell Type
R = 0.69
T−47D Importance
Ishikawa Importance
SIN3A Full Media Promoter
ER E2 Enhancer
Number Erbs
FOXA1 Enhancer
P300 Enhancer
P300 Promoter
MAX E2 Promoter
H3K27ac DMSO Promoter
SIN3A Stripped Media Promoter
ER DMSO Enhancer
JUN E2 Promoter
MAX DMSO Promoter
JUN DMSO Enhancer
RNAPII E2 Enhancer
H3K4me1 E2 Enhancer
RARA DMSO Promoter
H3K4me3 E2 Promoter
PRO Seq DMSO Enhancer
TAF1 E2 Promoter
RARA DMSO Enhancer
H3K4me1 DMSO Promoter
TAF1 DMSO Promoter
FOXA1 Promoter
TAF1 E2 Enhancer
JUN E2 Enhancer
ER DMSO Promoter
CTCF Promoter
MYC DMSO Promoter
DNase DMSO Promoter
Genelength
MYC E2 Enhancer
LSD1 E2 Promoter
GC Content Promoter
DNase E2 Promoter
MYC E2 Promoter
MAX E2 Enhancer
H3K27me3 E2 Enhancer
H3K27me3 E2 Promoter
shadowMean
H3K36me3 Promoter
H3K9me3 Promoter
H3K36me3 Enhancer
LSD1 DMSO Enhancer
shadowMin
H
Control
Early Up
Early Down
Late Up
Late Down
***
****
*
ns
−3 −2 −1 0 1 2
mRNA Half−Life (z−score by experiment)
Average of 54 measurements in 10 cell types

Supplementary Figure 3 (related to Figure 2). (A-D) Gene set enrichment of Early Up and Late Up genes for MSigDB Hallmark 2020 is shown for Ishikawa (A-B) and T-47D (C-D) cells. P-values are calculated using EnrichR. (E) Proportion of overlap between genes of each trajectory classification between cell types is shown. (F) Distribution in number of enhancers (z-score) by trajectory is plotted. Data is combined from both Ishikawa and T-47D cells. (G) The scatterplot shows median Boruta importance values for classifying trajectory in T-47D (y-axis) vs Ishikawa (x-axis). Boruta analysis was run separately in each cell type. Each data point represents a genomic feature. The correlation value was calculated using Pearson correlation. (H) mRNA half-life values from across 54 human samples were converted to Z-scores within each experiment. An average value was plotted for each gene, classified into trajectories based on the timing of the transcriptional response to E2. p-values are calculated using a Kolmogorov-Smirnov Test vs the Control group (ns > 0.05, * < 0.05, ** < 1e-5, *** < 1e-10, **** < 1e-15).

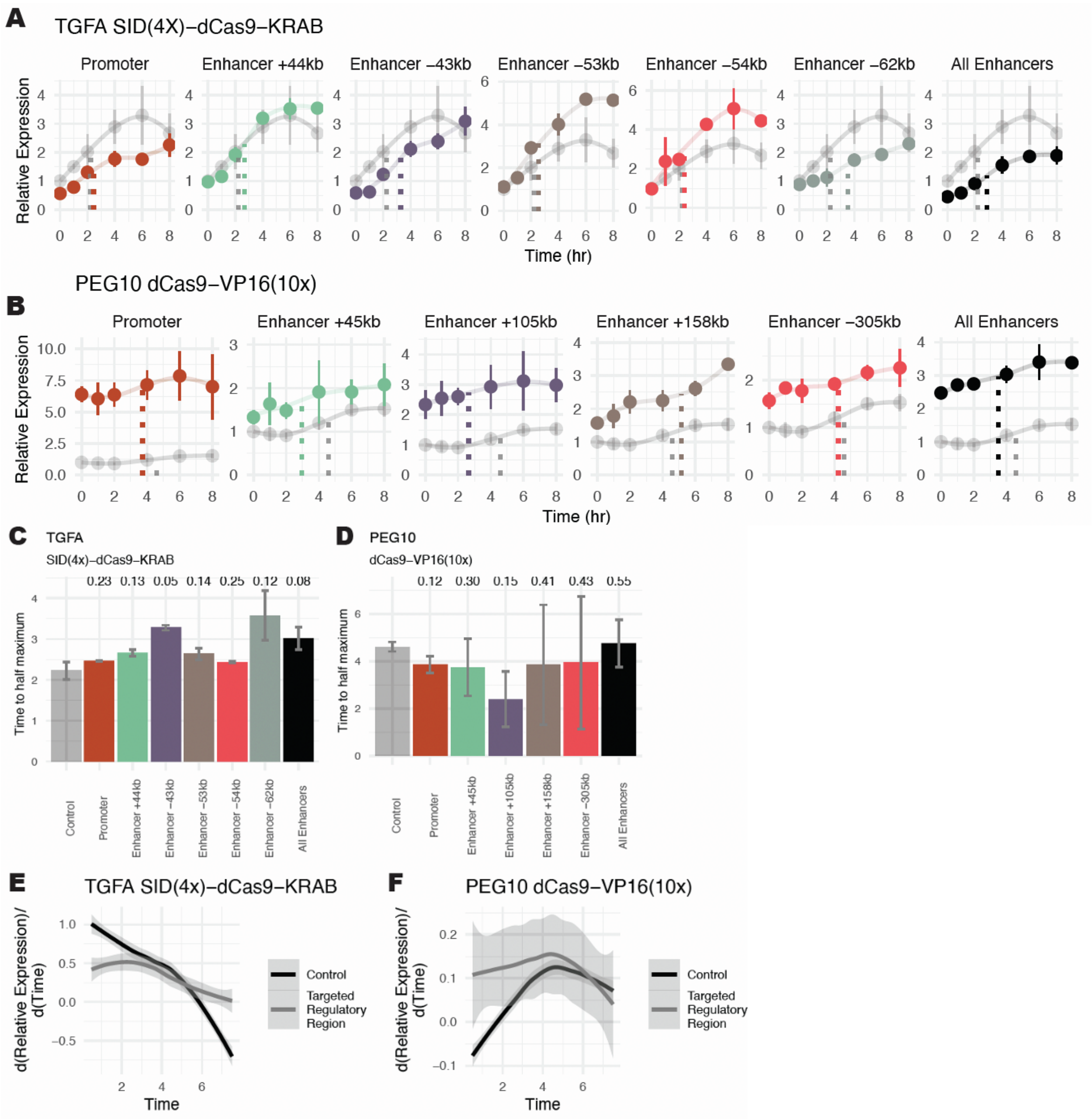
A
TGFA SID(4X)–dCas9–KRAB
Promoter
Enhancer +44kb
Enhancer –43kb
Enhancer –53kb
Enhancer –54kb
Enhancer –62kb
All Enhancers
Relative Expression
Time (hr)
B
PEG10 dCas9–VP16(10x)
Promoter
Enhancer +45kb
Enhancer +105kb
Enhancer +158kb
Enhancer –305kb
All Enhancers
Relative Expression
Time (hr)
C
TGFA
SID(4x)–dCas9–KRAB
0.23 0.13 0.05 0.14 0.25 0.12 0.08
Time to half maximum
Control
Promoter
Enhancer +44kb
Enhancer –43kb
Enhancer –53kb
Enhancer –54kb
Enhancer –62kb
All Enhancers
D
PEG10
dCas9–VP16(10x)
0.12 0.30 0.15 0.41 0.43 0.55
Time to half maximum
Control
Promoter
Enhancer +45kb
Enhancer +105kb
Enhancer +158kb
Enhancer –305kb
All Enhancers
E
TGFA SID(4x)–dCas9–KRAB
d(Relative Expression)/d(Time)
Time
Control
Targeted Regulatory Region
F
PEG10 dCas9–VP16(10x)
d(Relative Expression)/d(Time)
Time
Control
Targeted Regulatory Region

Supplementary Figure 4 (related to Figure 3). Synthetic transcription factors targeted to regulatory regions alter trajectories of *TGFA* and *PEG10*. (A) Expression trajectory of *TGFA* following E2 induction in Ishikawa cells is shown. Regulatory regions are targeted with SID(4x)-dCas9-KRAB. Data from cells with *IL1RN* promoter targeting (control) are shown in gray. Dotted lines represent the time to half maximal expression for each trajectory. Expression is relative to the 0-hour timepoint in the control. (B) *PEG10* gene expression trajectories in Ishikawa cells following E2 inductions are modulated by dCas9-VP16(10x) targeted to regulatory regions. Dotted lines represent the time to half maximal expression for each trajectory. Expression is relative to the 0-hour timepoint in the control. (C-D) Bar plot shows time to half maximal expression for each targeted regulatory region. Error bars represent the SEM (n=2) and p-values (one-sided t-test) are reported above each bar. (E-F) Aggregate differential of loess regressions from panels A and B show expression slope when CREs near *TGFA* are targeted by SID(4x)-dCas9-KRAB (E) or *PEG10* CREs are targeted by dCas9-VP16(10x) (F). Slopes are aggregated targeted (grey) compared to control (black) and the shaded region represents 95% confidence interval.

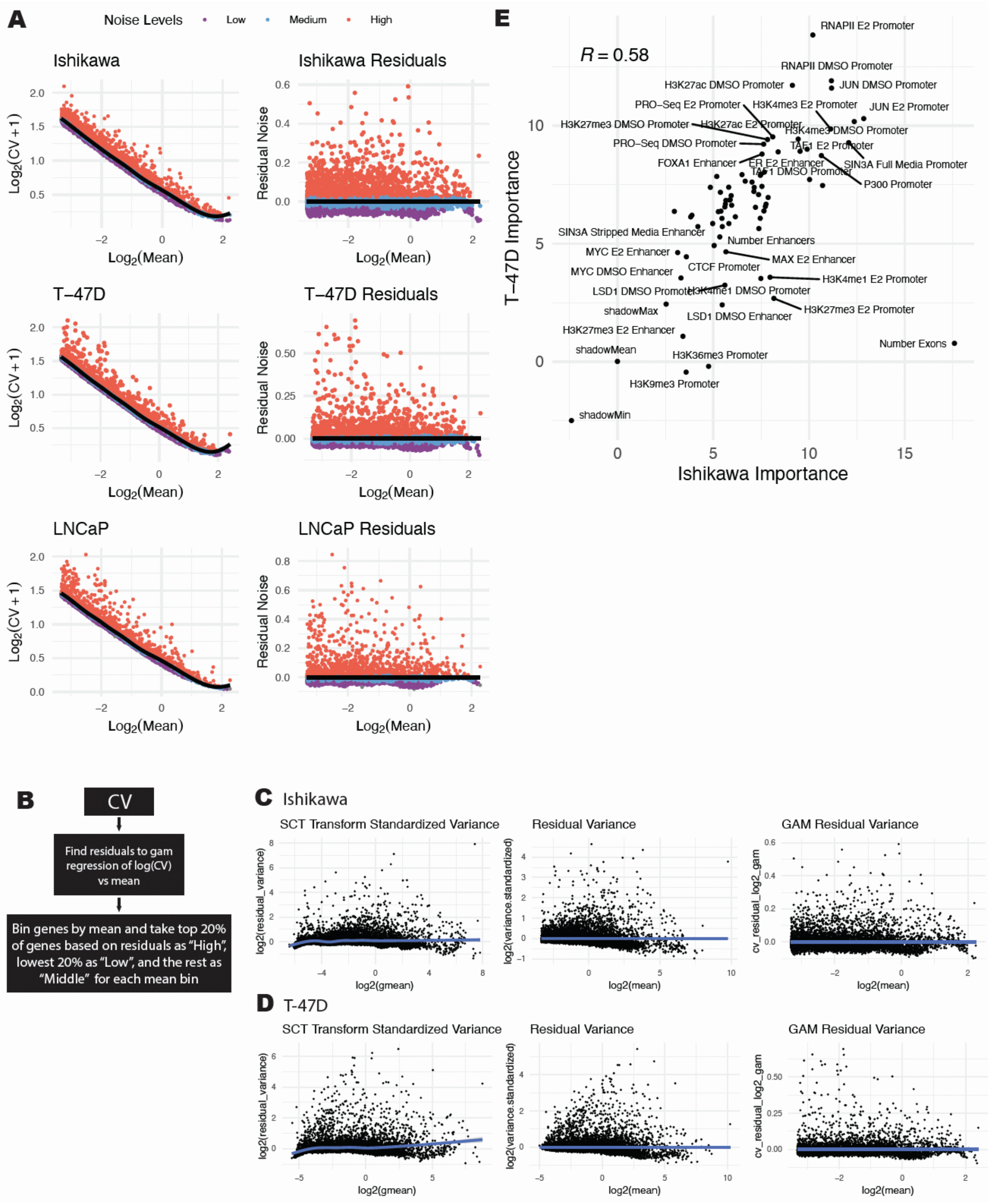
A
Noise Levels
Low
Medium
High
Ishikawa
Ishikawa Residuals
T−47D
T−47D Residuals
LNCaP
LNCaP Residuals
Log2(CV + 1)
Residual Noise
Log2(Mean)
E
R = 0.58
T−47D Importance
Ishikawa Importance
RNAPII E2 Promoter
RNAPII DMSO Promoter
H3K27ac DMSO Promoter
JUN DMSO Promoter
JUN E2 Promoter
PRO−Seq E2 Promoter
H3K4me3 E2 Promoter
H3K27me3 DMSO Promoter
H3K27ac E2 Promoter
H3K4me3 DMSO Promoter
PRO−Seq DMSO Promoter
TAF1 E2 Promoter
FOXA1 Enhancer
ER E2 Enhancer
SIN3A Full Media Promoter
TAF1 DMSO Promoter
P300 Promoter
SIN3A Stripped Media Enhancer
Number Enhancers
MYC E2 Enhancer
MAX E2 Enhancer
CTCF Promoter
MYC DMSO Enhancer
H3K4me1 E2 Promoter
LSD1 DMSO Promoter
H3K4me1 DMSO Promoter
shadowMax
H3K27me3 E2 Promoter
LSD1 DMSO Enhancer
H3K27me3 E2 Enhancer
shadowMean
Number Exons
H3K36me3 Promoter
H3K9me3 Promoter
shadowMin
B
CV
Find residuals to gam regression of log(CV) vs mean
Bin genes by mean and take top 20% of genes based on residuals as "High", lowest 20% as "Low", and the rest as "Middle" for each mean bin
C Ishikawa
D T-47D
SCT Transform Standardized Variance
Residual Variance
GAM Residual Variance
log2(residual_variance)
log2(variance.standardized)
cv_residual_log2_gam
log2(gmean)
log2(mean)

Supplementary Figure 5 (related to Figure 4). Strong enhancers and weak promoters are associated with high levels of noise. (A) Calculated coefficient of variation (CV) is shown before (left panel) and after (right panel) adjustment using a generalized additive model regression fit for each cell type. Axes are on a log scale. Colors represent classified noise levels. (B) A flowchart depicts the regularization strategy to remove mean effects from the measured variation and to classify genes into noise levels. (C-D) A comparison of noise regularization strategies in (C) Ishikawa and (D) T-47D cells is shown. SCT transform standardized variance and residual variance were calculated using the Seurat package in R. Blue line shows a loess best fit. (E) Boruta importance scores for classifying genes into noise categories are shown for independent analyses in T-47D (y-axis) and Ishikawa (x-axis) cells. Correlation value was calculated using Pearson correlation.

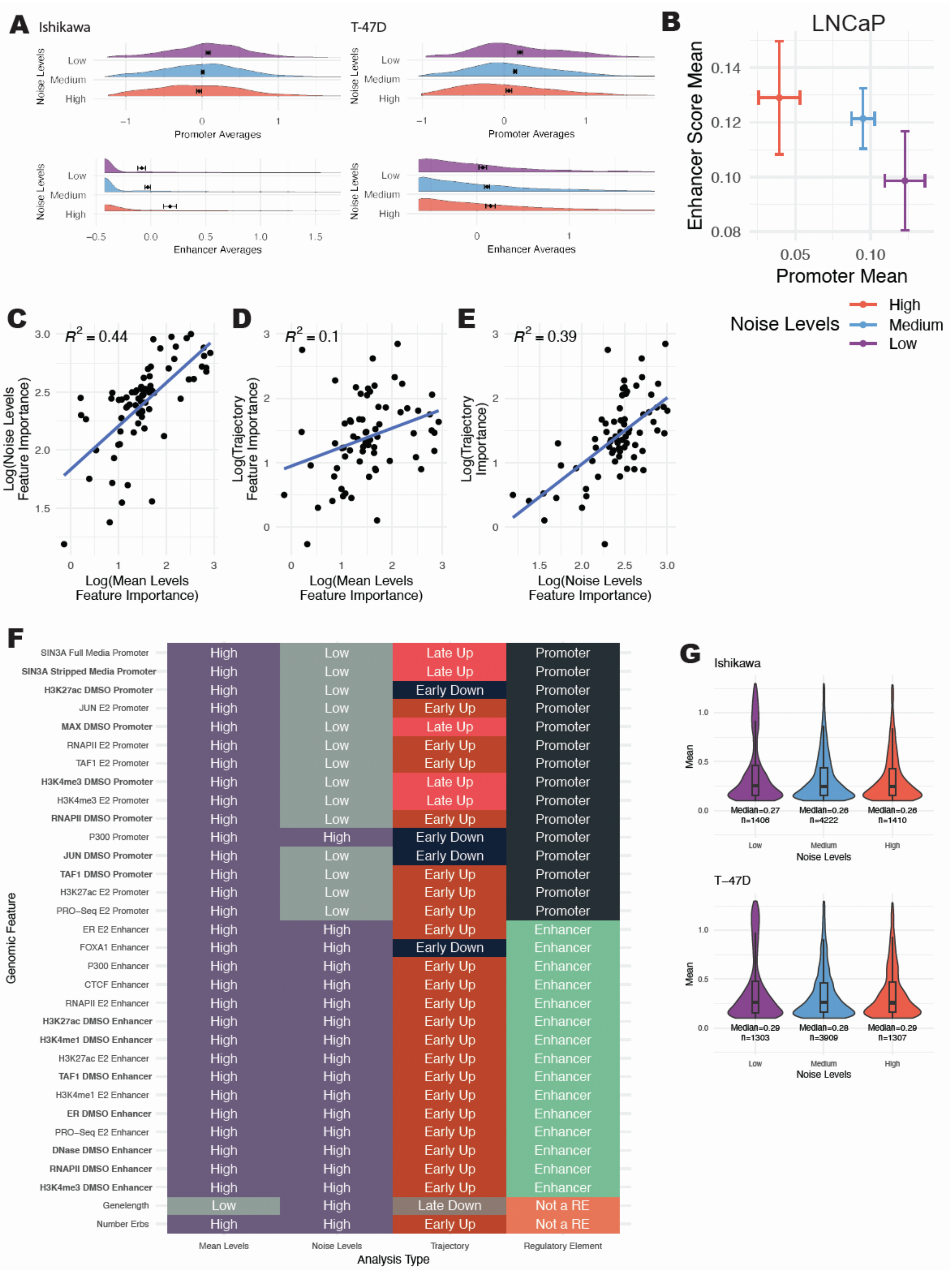

A
Ishikawa
T-47D
Noise Levels
Low
Medium
High
Promoter Averages
Enhancer Averages
B
LNCaP
Enhancer Score Mean
Promoter Mean
Noise Levels
High
Medium
Low
C
$R^2 = 0.44$
Log(Noise Levels Feature Importance)
Log(Mean Levels Feature Importance)
D
$R^2 = 0.1$
Log(Trajectory Feature Importance)
Log(Mean Levels Feature Importance)
E
$R^2 = 0.39$
Log(Trajectory Importance)
Log(Noise Levels Feature Importance)
F
Genomic Feature
Analysis Type
Mean Levels
Noise Levels
Trajectory
Regulatory Element
SIN3A Full Media Promoter
SIN3A Stripped Media Promoter
H3K27ac DMSO Promoter
JUN E2 Promoter
MAX DMSO Promoter
RNAPII E2 Promoter
TAF1 E2 Promoter
H3K4me3 DMSO Promoter
H3K4me3 E2 Promoter
RNAPII DMSO Promoter
P300 Promoter
JUN DMSO Promoter
TAF1 DMSO Promoter
H3K27ac E2 Promoter
PRO–Seq E2 Promoter
ER E2 Enhancer
FOXA1 Enhancer
P300 Enhancer
CTCF Enhancer
RNAPII E2 Enhancer
H3K27ac DMSO Enhancer
H3K4me1 DMSO Enhancer
H3K27ac E2 Enhancer
TAF1 DMSO Enhancer
H3K4me1 E2 Enhancer
ER DMSO Enhancer
PRO–Seq E2 Enhancer
DNase DMSO Enhancer
RNAPII DMSO Enhancer
H3K4me3 DMSO Enhancer
Genelength
Number Erbs
High
Low
Late Up
Early Up
Early Down
Late Down
Promoter
Enhancer
Not a RE
G
Ishikawa
T–47D
Mean
Noise Levels
Median=0.27 n=1406
Median=0.26 n=4222
Median=0.26 n=1410
Median=0.29 n=1303
Median=0.28 n=3909
Median=0.29 n=1307

Supplementary Figure 6 (related to Figure 5). Promoters and enhancers underlie different relationships between mean, noise, and trajectory. (A) The full distribution of promoter (top) and enhancer (bottom) in Ishikawa (left) and T-47D (right) across noise levels is shown. (B) Enhancer score averages vs promoter score averages are shown for noise levels in LNCaP cells. Averages are based on confirmed variables from a Boruta analysis. Axes do not represent full range. For a list of genomic data used in LNCaP cells see Table S3. (C-E) Pairwise correlation plots show the relationship between importance scores for the three analysis types. Each point represents a genomic feature. R^2 values were calculated using Spearman correlation. (F) Top 15 most important features for each regulatory element type (promoter or enhancer) are shown, determined by average rank across all three analysis types. For each analysis, the classification group with the highest signal is displayed. Datasets shown in bold were performed in the absence of ER activation. (G) Graph depicts mean levels across different noise levels for Ishikawa (top) and T-47D (bottom).

Table S1. gRNA sequences, related to STAR Methods

gRNA Name	Sequence	PAM
PEG10 Promoter gRNA 1	GCCTTATATAGGTTGCACCG	AGG
PEG10 Promoter gRNA 2	CGCGTGGTGAGTATGCGGTG	AGG
PEG10 Promoter gRNA 3	GGAGTACGGGATTACCGAGG	GGG
PEG10 Promoter gRNA 4	TTAGTGGCCTGCGCGCGACG	TGG
PEG10 Enhancer +105kb gRNA 1	CATCTCCAGTTCAAACAGTG	AGG
PEG10 Enhancer +105kb gRNA 2	AAGCCCAGGTATATTAATGG	AGG
PEG10 Enhancer +105kb gRNA 3	TGACATAAACTAGCTGAGCA	GGG
PEG10 Enhancer +105kb gRNA 4	ACTTGAGCTTGGATCCAGCT	GGG
PEG10 Enhancer +158kb gRNA 1	GAGAGTTCCACTCTCGTGAA	TGG
PEG10 Enhancer +158kb gRNA 2	GACAGAGCAAGACTCCGTAA	AGG
PEG10 Enhancer +158kb gRNA 3	ATCTGCTGGAACCTTGATCA	TGG
PEG10 Enhancer +158kb gRNA 4	GAGGAGAGCCTCTTGAACCT	GGG
PEG10 Enhancer +45kb gRNA 1	GGAATATGCTCATAACCACA	AGG
PEG10 Enhancer +45kb gRNA 2	CTCTTAATCTCCCTTCACCA	AGG
PEG10 Enhancer +45kb gRNA 3	TCCTATTTCCTGTTGCCCTG	GGG
PEG10 Enhancer +45kb gRNA 4	CCTAATTCCAGGCTCACCAC	AGG
PEG10 Enhancer +-305kb gRNA 1	GGCATACTGGAATAAACATG	AGG
PEG10 Enhancer +-305kb gRNA 2	TGAGAGACTCACTTGTCACT	AGG
PEG10 Enhancer +-305kb gRNA 3	ACACTTCTAGAAGGCCCACT	AGG
PEG10 Enhancer +-305kb gRNA 4	TAAAGAAGGTATAAGATCCA	TGG
TACSTD2 Promoter gRNA 1	GGTAGAGTATAAGAGCCGGA	GGG
TACSTD2 Promoter gRNA 2	TGTTCTGATCCTATCGCGGG	CGG
TACSTD2 Promoter gRNA 3	GAGAACCGATACTCACCTGT	AGG
TACSTD2 Promoter gRNA 4	TAACACCAGTCCTGACAGGT	AGG
TACSTD2 Enhancer +4.7kb gRNA 1	CATCAGGAGACTAACCAGGG	AGG
TACSTD2 Enhancer +4.7kb gRNA 2	GAAGACACTGAAGCTCATAG	AGG
TACSTD2 Enhancer +4.7kb gRNA 3	AAGCCCTGGTGACCTACACA	AGG
TACSTD2 Enhancer +4.7kb gRNA 4	CAGTGAACCAGTGAACTGTA	AGG
TACSTD2 Enhancer -7.1kb gRNA 1	TGTAAGGGCTTTACACTCTG	GGG
TACSTD2 Enhancer -7.1kb gRNA 2	AGCAGAACTGTACACATCAC	AGG
TACSTD2 Enhancer -7.1kb gRNA 3	GCACCGTGACTCATCAGTGT	GGG
TACSTD2 Enhancer -7.1kb gRNA 4	GATGTGTGATGCCCTGTTAA	GGG
TACSTD2 Enhancer -15.2kb gRNA 1	TGGATTCAAGATCGAAACCC	TGG
TACSTD2 Enhancer -15.2kb gRNA 2	TGACTTCATCAGATGGAAAG	TGG
TACSTD2 Enhancer -15.2kb gRNA 3	GACATGAACGAGTCCTTGGA	AGG
TACSTD2 Enhancer -15.2kb gRNA 4	TGGGCAGGCTGATTAATGAG	GGG
TGFA Enhancer +44kb gRNA 1	TGAGTACCCAGCTCATGCA	AGG

TGFA Enhancer +44kb gRNA 2	GGGCATTACTCAGGCGCCC	GGG
TGFA Enhancer +44kb gRNA 3	CCCTGGTTAAGAAGTTGTT	GGG
TGFA Enhancer +44kb gRNA 4	GACAGGTGTTCTAAGCTAT	AGG
TGFA Promoter gRNA 1	CCCCGGTCGCCGAGTGGCG	AGG
TGFA Promoter gRNA 2	GAGCGCACCGAGCAGGGCG	CGG
TGFA Promoter gRNA 3	GCACGGCGCTGGCAGTCGG	GGG
TGFA Promoter gRNA 4	TAGGTAACGGGTGCCCGGG	CGG
TGFA Enhancer -43kb gRNA 1	GGAAGCGGGATGCAGACCT	GGG
TGFA Enhancer -43kb gRNA 2	CAAGACATCAGGCTTCCTG	GGG
TGFA Enhancer -43kb gRNA 3	GTTCACCACAGCGTGTCAA	GGG
TGFA Enhancer -43kb gRNA 4	CCAGGATCTGCCACCCCTG	TGG
TGFA Enhancer -53kb gRNA 1	CACCGAATCAACTGAGGTC	AGG
TGFA Enhancer -53kb gRNA 2	GGAGTTCCTACAATCACAG	AGG
TGFA Enhancer -53kb gRNA 3	AAGACAACGGATGTGTCCG	TGG
TGFA Enhancer -53kb gRNA 4	AGAAGCCCGAACCCAGAGA	AGG
TGFA Enhancer -54kb gRNA 1	GAACGGGGACTTGAGGTAT	TGG
TGFA Enhancer -54kb gRNA 2	AGCATCACGCTGAGAATGG	GGG
TGFA Enhancer -54kb gRNA 3	GGACATCCACTTGAAAAGC	TGG
TGFA Enhancer -54kb gRNA 4	TAAAATGAGTGCTCTAGGC	AGG
TGFA Enhancer -62kb gRNA 1	AGGGCTACCTAACTGCAAT	AGG
TGFA Enhancer -62kb gRNA 2	ATTGTCTGAACTCCGGATG	TGG
TGFA Enhancer -62kb gRNA 3	TCAGGGAGTGTTGCAACCT	TGG
TGFA Enhancer -62kb gRNA 4	GTATCACAGCGAAGCGGGT	GGG

Table S2. qPCR primers, related to STAR Methods

Primer name	Sequence
TACSTD2_F	ACAACGATGGCCTCTACGAC
TACSTD2_R	GTCCAGGTCTGAGTGGTTGAA
PEG10_F	GAGCACCAGGGATTTCTCAGT
PEG10_R	GGTAGTTGTGCATCAGGTAGTG
TGFA_F	AGGTCCGAAAACACTGTGAGT
TGFA_R	AGCAAGCGGTTCTTCCCTTC
IL1RN_F	GGAATCCATGGAGGGAAGAT
IL1RN_R	TGTTCTCGCTCAGGTCAGTG
dCas9_F	GTGACCGAGGGAATGAGAAA
dCas9_R	AGCTGCTTCACGGTCACTTT

Table S3. Genomic Source Information, related to STAR Methods
* - indicates experiments conducted for this study. See Data Availability.

Cell Line	Genomic Feature	Reference	Accession
Ishikawa	CTCF	[S1]	GSE32465
Ishikawa	Dnase	[S1]	GSE32970
Ishikawa	ER	[S1,S2]	GSE32465; GSE129803
Ishikawa	FOXA1	[S1]	GSE32465
Ishikawa	GC Content	[S3]	
Ishikawa	Gene length	[S3]	
Ishikawa	H3K27ac	[S4]	GSE109893
Ishikawa	H3K27me3	*	
Ishikawa	H3K36me3	*	
Ishikawa	H3K4me1	*	
Ishikawa	H3K4me3	*	
Ishikawa	H3K9me3	[S5]	GSE99906
Ishikawa	JUN	*	
Ishikawa	LSD1	*	
Ishikawa	MAX	*	
Ishikawa	MYC	*	
Ishikawa	Number Enhancers	*	
Ishikawa	Number ERBS	*	
Ishikawa	p300	[S1]	GSE32465
Ishikawa	PRO-seq	*	
Ishikawa	RARA	*	
Ishikawa	RNAPII	[S1,S5]	GSE32465; GSE99906
Ishikawa	SIN3A	*	

Ishikawa	TAF1	*	
Ishikawa	scRNA-seq	*	
Ishikawa	HiChIP	*	
T-47D	CTCF	[S1]	GSE32465
T-47D	Dnase	[S1]	GSE32970
T-47D	ER	[S1]	GSE32465
T-47D	FOXA1	[S1]	GSE32465
T-47D	GC Content	[S3]	
T-47D	Gene length	[S3]	
T-47D	H3K27ac	[S6,S7]	GSE63109; GSE147141
T-47D	H3K27me3	*	
T-47D	H3K36me3	[S8]	GSE63109
T-47D	H3K4me1	*	
T-47D	H3K4me3	*	
T-47D	H3K9me3	[S8]	GSE63109
T-47D	JUN	*	
T-47D	LSD1	*	
T-47D	MAX	*	
T-47D	MYC	*	
T-47D	Number Enhancers	*	
T-47D	Number ERBS	*	
T-47D	p300	[S1]	GSE32465
T-47D	PRO-seq	*	
T-47D	RARA	*	
T-47D	RNAPII	*	
T-47D	SIN3A	*	
T-47D	TAF1	*	
T-47D	scRNA-seq	*	
T-47D	HiChIP	*	
LNCaP	H3ac	[S9,S10]	GSE62492
LNCaP	AR	[S9,S10]	GSE62492
LNCaP	CHD1	[S11]	GSE64530
LNCaP	ETV1	[S12]	GSE47120
LNCaP	FOXP1	[S9,S10]	GSE62492
LNCaP	H3K27ac	[S13]	GSE73785
LNCaP	H3K27me3	[S9,S10]	GSE62492
LNCaP	H3K4me1	[S9,S10]	GSE62492
LNCaP	H3K4me3	[S9,S10]	GSE62492
LNCaP	LSD1	[S11]	GSE64530

LNCaP	RUNX1	[S9,S10]	GSE62492
LNCaP	scRNA-seq	[S14]	GSE168668
LNCaP	HiChIP	*	

Supplemental References

S1. ENCODE, C. (2012). An integrated encyclopedia of DNA elements in the human genome. Nature *489*, 57-74. 10.1038/nature11247.

S2. Rodriguez, A.C., Vahrenkamp, J.M., Berrett, K.C., Clark, K.A., Guillen, K.P., Scherer, S.D., Yang, C.-H., Welm, B.E., Janát-Amsbury, M.M., and Graves, B.J. (2020). ETV4 is necessary for estrogen signaling and growth in endometrial cancer cells. Cancer research *80*, 1234-1245.

S3. Kent, W.J., Sugnet, C.W., Furey, T.S., Roskin, K.M., Pringle, T.H., Zahler, A.M., and Haussler, D. (2002). The human genome browser at UCSC. Genome research *12*, 996-1006.

S4. Vahrenkamp, J.M., Yang, C.H., Rodriguez, A.C., Almomen, A., Berrett, K.C., Trujillo, A.N., Guillen, K.P., Welm, B.E., Jarboe, E.A., Janat-Amsbury, M.M., and Gertz, J. (2018). Clinical and Genomic Crosstalk between Glucocorticoid Receptor and Estrogen Receptor α In Endometrial Cancer. Cell Rep *22*, 2995-3005. 10.1016/j.celrep.2018.02.076.

S5. Carleton, J.B., Berrett, K.C., and Gertz, J. (2017). Multiplex Enhancer Interference Reveals Collaborative Control of Gene Regulation by Estrogen Receptor α-Bound Enhancers. Cell Syst *5*, 333-344.e335. 10.1016/j.cels.2017.08.011.

S6. Zhang, G., Zhao, Y., Liu, Y., Kao, L.P., Wang, X., Skerry, B., and Li, Z. (2016). FOXA1 defines cancer cell specificity. Sci Adv *2*, e1501473. 10.1126/sciadv.1501473.

S7. Carleton, J.B., Ginley-Hidinger, M., Berrett, K.C., Layer, R.M., Quinlan, A.R., and Gertz, J. (2020). Regulatory sharing between estrogen receptor α bound enhancers. Nucleic Acids Research *48*, 6597-6610. 10.1093/nar/gkaa454.

S8. Zhang, G., Zhao, Y., Liu, Y., Kao, L.-P., Wang, X., Skerry, B., and Li, Z. (2016). FOXA1 defines cancer cell specificity. Science Advances *2*, e1501473. doi:10.1126/sciadv.1501473.

S9. Takayama, K., Suzuki, T., Tsutsumi, S., Fujimura, T., Urano, T., Takahashi, S., Homma, Y., Aburatani, H., and Inoue, S. (2015). RUNX1, an androgen- and EZH2-regulated gene, has differential roles in AR-dependent and -independent prostate cancer. Oncotarget *6*, 2263-2276. 10.18632/oncotarget.2949.

S10. Yamamoto, S., Takayama, K.i., Obinata, D., Fujiwara, K., Ashikari, D., Takahashi, S., and Inoue, S. (2019). Identification of new octamer transcription factor 1-target genes upregulated in castration-resistant prostate cancer. Cancer Science *110*, 3476-3485.

S11. Metzger, E., Willmann, D., McMillan, J., Forne, I., Metzger, P., Gerhardt, S., Petroll, K., Von Maessenhausen, A., Urban, S., and Schott, A.-K. (2016). Assembly of methylated KDM1A and CHD1 drives androgen receptor–dependent transcription and translocation. Nature structural & molecular biology *23*, 132-139.

S12. Chen, Y., Chi, P., Rockowitz, S., Iaquinta, P.J., Shamu, T., Shukla, S., Gao, D., Sirota, I., Carver, B.S., and Wongvipat, J. (2013). ETS factors reprogram the androgen receptor cistrome and prime prostate tumorigenesis in response to PTEN loss. Nature medicine *19*, 1023-1029.

S13. Taberlay, P.C., Achinger-Kawecka, J., Lun, A.T., Buske, F.A., Sabir, K., Gould, C.M., Zotenko, E., Bert, S.A., Giles, K.A., and Bauer, D.C. (2016). Three-dimensional disorganization of the cancer genome occurs coincident with long-range genetic and epigenetic alterations. Genome research *26*, 719-731.

S14. Taavitsainen, S., Engedal, N., Cao, S., Handle, F., Erickson, A., Prekovic, S., Wetterskog, D., Tolonen, T., Vuorinen, E.M., Kiviaho, A., et al. (2021). Single-cell ATAC and RNA sequencing reveal pre-existing and persistent cells associated with prostate cancer relapse. Nat Commun *12*, 5307. 10.1038/s41467-021-25624-1.
